# Effects of Dietary *Rhodotorula mucilaginosa* Powder Supplementation on Growth, Coloration, and Health in Fancy Carp (*Cyprinus carpio* Linn.)

**DOI:** 10.3390/ani16162468

**Published:** 2026-08-08

**Authors:** Worrawat Promden, Eakapol Wangkahart, Nantaporn Sutthi, Wassana Prisingkorn, Janjira Phudkliang, Wilailak Ounjit, Ruamruedee Panchan

**Affiliations:** 1 Division of General Science, Faculty of Education, Buriram Rajabhat University, Buriram 31000, Thailand; worrawat.pd@bru.ac.th; 2 Applied Animal and Aquatic Sciences Research Unit, Division of Fisheries, Faculty of Technology, Mahasarakham University, Maha Sarakham 44150, Thailand; eakapol.w@msu.ac.th (E.W.); nantaporn175sutthi@gmail.com (N.S.); janjiphurrt@gmail.com (J.P.); 3 Aquatic Animal Disease and Molecular Biology Laboratory, Department of Fisheries, Faculty of Agriculture, Khon Kaen University, Khon Kaen 40002, Thailand; wasspr@kku.ac.th; 4 Department of Sociology and Anthropology, Faculty of Humanities and Social Sciences, Mahasarakham University, Maha Sarakham 44150, Thailand; wilailak.o@msu.ac.th

**Keywords:** *Rhodotorula mucilaginosa*, *Cyprinus carpio*, feed additive, carotenoid deposition, fish health

## Abstract

Dietary carotenoids and functional feed additives are increasingly used in ornamental aquaculture to improve fish coloration, growth performance, and health. This study evaluated the effects of dietary *Rhodotorula mucilaginosa* powder on growth performance, feed utilization, pigmentation, physiological responses, hepatic gene expression, and tissue morphology in fancy carp (*Cyprinus carpio* Linn.). Fish were fed five isonitrogenous and isoenergetic diets containing 0, 0.5, 1, 2, or 4 g/kg *R. mucilaginosa* powder for 70 days. Fish fed 0.5 g/kg achieved the highest weight gain (25.38 ± 1.56 g) and average daily gain (0.36 ± 0.02 g/day), while 0.5 and 1 g/kg significantly improved feed conversion ratio (1.63 ± 0.03 and 1.70 ± 0.04, respectively) compared with the control and higher inclusion levels (*p* < 0.05). Supplementation with 2 g/kg produced the greatest skin redness (CIE a* = 17.94 ± 0.34) and skin carotenoid content (15.62 ± 0.89 µg/g) (*p* < 0.05). Lower inclusion levels also significantly improved intestinal morphology and upregulated hepatic *CAT*, *SOD*, *IL-1β*, *VTG*, and *HSP70* expression (*p* < 0.05), whereas serum biochemical parameters, cortisol concentration, survival, and liver histopathology remained unaffected (*p* > 0.05). Overall, *R. mucilaginosa* powder is a safe multifunctional feed additive. Dietary inclusion of 0.5–1 g/kg is recommended to optimize growth and feed efficiency, whereas 2 g/kg is optimal for enhancing pigmentation. These findings support the development of sustainable natural pigment-based functional feeds for ornamental aquaculture.

## Simple Summary

Fancy carp require diets that support coloration, growth, and health. This study evaluated the dietary *Rhodotorula mucilaginosa* powder as a natural feed additive over 70 days. Supplementation at 0.5–1 g/kg improved feed efficiency and growth, while 2 g/kg produced the greatest enhancement in red coloration and skin carotenoid content. Survival, blood health, and stress indicators were unaffected, and no major tissue abnormalities were observed. These results demonstrate that *R. mucilaginosa* powder is a safe and promising natural alternative to synthetic pigments in ornamental fish diets.

## 1. Introduction

The ornamental fish industry has expanded rapidly over the past two decades, driving increasing demand for fish exhibiting superior coloration, growth performance, and health status [1,2]. Among ornamental species, fancy carp (*Cyprinus carpio*) are particularly valued for their distinctive pigmentation patterns and aesthetic appeal, with body coloration serving as a major determinant of market value and consumer preference [3,4]. However, maintaining vibrant and stable pigmentation remains a major challenge because fish cannot synthesize carotenoids de novo and therefore rely entirely on dietary sources for pigment deposition [5]. Consequently, improving carotenoid availability and utilization has become a central objective in ornamental aquaculture nutrition [6,7]. Beyond their role in pigmentation, carotenoids are increasingly recognized as multifunctional bioactive compounds that regulate antioxidant defense, immune competence, reproduction, growth, and overall physiological health [8,9,10].

Synthetic carotenoids, particularly astaxanthin and canthaxanthin, remain the most widely used pigment sources because of their high coloring efficiency and consistent quality [8,11]. Nevertheless, their commercial application is increasingly constrained by high production costs, dependence on chemical synthesis, and growing consumer and regulatory preferences for naturally derived and environmentally sustainable alternatives [12,13,14]. Consequently, considerable attention has shifted toward natural carotenoid sources capable of simultaneously improving pigmentation and fish health [15,16,17,18,19]. Plant-derived ingredients, including mulberry, marigold flowers, sesame seeds, dragon fruit peel, carrot, and ginger, have demonstrated promising effects on pigmentation and selected physiological traits in ornamental fish [20,21,22,23,24,25]. However, their efficacy remains inconsistent because carotenoid composition, bioavailability, digestibility, and absorption efficiency vary substantially among plant species and fish taxa [26,27,28,29]. In addition, the large-scale production of plant-derived carotenoids is constrained by seasonal availability, geographic variation, relatively low pigment yields, and the requirement for substantial plant biomass, thereby reducing their economic competitiveness in commercial aquaculture [30,31].

To overcome these limitations, microbially derived feed additives have emerged as promising alternatives because they integrate nutritional, probiotic, immunological, and functional properties within a single ingredient [32,33,34,35,36]. Among these, yeast-based feed additives have attracted considerable attention owing to their rich content of carotenoids, β-glucans, mannan-oligosaccharides, nucleotides, vitamins, and functional proteins, which enhance nutrient utilization and host physiology [34,37]. A comprehensive review by Mohammed et al. [36], which synthesized evidence from more than 150 aquaculture studies, concluded that *Saccharomyces cerevisiae* consistently improves growth, feed efficiency, immunity, and intestinal health, although responses vary with yeast strain, dietary inclusion level, host species, and culture conditions. Supporting these findings, dietary *S. cerevisiae* and yeast-derived prebiotics improved intestinal morphology in common carp, including villus dimensions and muscular thickness, despite having no significant effect on growth under the experimental conditions [38]. Similar benefits have also been reported for *S. cerevisiae*, yeast hydrolysates, and *Sporidiobolus pararoseus*, which enhance growth, intestinal morphology, antioxidant capacity, immunity, and disease resistance [39,40,41,42]. In addition, carotenoid-rich *Haematococcus pluvialis* and *Phaffia rhodozyma* improve pigmentation, carotenoid deposition, and antioxidant status [43,44,45,46]. Collectively, these findings highlight the multifunctional benefits of microbial feed additives beyond pigmentation.

Among pigment-producing yeasts, *Rhodotorula mucilaginosa* has attracted increasing attention because of its exceptional capacity to synthesize β-carotene, torulene, and torularhodin while simultaneously producing β-glucans, mannan-oligosaccharides, lipids, and other bioactive metabolites with recognized nutritional and immunomodulatory properties [47,48,49,50]. These bioactive components have been associated with improved digestive efficiency, intestinal integrity, antioxidant defense, immune competence, pigmentation, and water quality [51,52,53,54,55], leading to the recognition of *Rhodotorula* spp. as multifunctional feed additives in aquaculture [51,56,57,58,59,60,61,62,63,64,65,66]. Experimental evidence further supports these functional attributes. Dietary *R. mucilaginosa* supplementation has enhanced growth performance, antioxidant capacity, immune responses, digestive enzyme activity, intestinal morphology, and disease resistance in Pacific white shrimp, Nile tilapia, Atlantic salmon, golden pompano, and red claw crayfish [67,68,69,70,71,72,73,74]. In addition, its pigment-enhancing capacity has recently been demonstrated in gilthead seabream and ornamental koi carp [75,76], highlighting its potential as a sustainable alternative to synthetic carotenoids.

Despite this growing body of evidence, important knowledge gaps remain. Most studies on yeast-based feed additives have focused on food fish, shrimp, or single physiological endpoints, whereas comprehensive dose–response evaluations integrating growth, pigmentation, digestive physiology, antioxidant and immune responses, molecular regulation, and tissue morphology in ornamental fish remain scarce. In particular, the optimal dietary inclusion level of *R. mucilaginosa* powder for maximizing both pigmentation and physiological performance in fancy carp has yet to be established, and the relationships among intestinal adaptations, hepatic molecular responses, and pigmentation remain poorly understood. Addressing these gaps is essential for developing scientifically validated functional feeds that simultaneously improve fish health and commercial quality.

Therefore, the aims of this study were to evaluate the dose-dependent effects of dietary *R. mucilaginosa* powder supplementation on growth performance, feed utilization, body coloration, carotenoid accumulation, blood biochemical parameters, antioxidant and immune responses, digestive enzyme activities, gene expression, and intestinal and hepatic histomorphology in fancy carp (*C. carpio*), and to determine the optimal dietary supplementation level for improving fish performance and pigmentation. We hypothesized that *R. mucilaginosa* functions as a multifunctional feed additive capable of enhancing pigmentation while simultaneously improving physiological health through the coordinated modulation of digestive, antioxidant, immune, and intestinal pathways. The findings provide scientific evidence supporting the development of sustainable functional feeds for ornamental fish and help identify an appropriate dietary supplementation level for improving both fish health and commercial quality.

## 2. Materials and Methods

### 2.1. Preparation and Characterization of R. mucilaginosa Powder

A strain of *R. mucilaginosa* (formerly *R. rubra*; TISTR No. 5067) was obtained from the TISTR Culture Collection (Thailand Institute of Scientific and Technological Research, Thailand) and maintained on Yeast Malt (YM) agar. The yeast was cultivated in modified YM broth containing peptic digest of animal tissue (5.0 g/L), yeast extract (3.0 g/L), malt extract (3.0 g/L), and sucrose (30.0 g/L) at pH 3.8 ± 0.2. Following sterilization (121 °C, 15 min), a starter culture was incubated at 25 °C and 200 rpm for 24 h before inoculating fresh medium (10%, *v*/*v*) for 7 days under the same conditions. All cultures were prepared in triplicate. Biomass was harvested by centrifugation (5000× *g*, 10 min), washed twice with sterile distilled water, frozen at −20 °C for at least 24 h, freeze-dried (24 h primary drying followed by 3 h secondary drying at 30 °C), finely ground, and stored in light-proof containers at −20 °C until use. Viable cell concentrations before and after freeze-drying were determined by the standard plate count method following serial dilution in 0.85% NaCl and incubation on YM agar at 25 °C for 48 h. Only plates containing 30–300 colonies were enumerated. The freeze-dried biomass contained 2.0 × 10^13^ CFU/g dry weight.

Total carotenoid in the freeze-dried biomass was quantified using a modified spectrophotometric method based on Carvalho et al. [77]. Briefly, samples were extracted with acetone, dimethyl sulfoxide, and petroleum ether, followed by ultrasonication and centrifugation. Absorbance was measured at 450 nm using a GENESYS™ 10S UV–Vis spectrophotometer (Thermo Fisher Scientific, Waltham, MA, USA), and carotenoid concentration was calculated according to the Beer–Lambert equation using the specific extinction coefficient of β-carotene in petroleum ether (A_1%,1 cm_ = 2592). All analyses were performed in quadruplicate, and total carotenoid content of the freeze-dried *R. mucilaginosa* biomass was 30.57 ± 3.81 µg/g dry weight.

### 2.2. Experimental Diets and Proximate Composition

The dietary inclusion levels of *R. mucilaginosa* (0.5, 1, 2, and 4 g/kg diet) were selected based on previous studies investigating *Rhodotorula* spp. and other yeast-derived functional feed additives in aquaculture, which reported beneficial effects on growth performance, pigmentation, antioxidant capacity, and immune responses within a similar supplementation range while suggesting limited additional benefits at higher inclusion levels [61]. Accordingly, these concentrations were chosen to represent low, moderate, and relatively high dietary supplementation levels to establish the optimal inclusion rate for fancy carp. Five isonitrogenous and isoenergetic experimental diets were formulated to contain graded levels of *R. mucilaginosa* powder at 0 (RM0), 0.5 (RM0.5), 1 (RM1), 2 (RM2), and 4 g/kg diet (RM4). The yeast powder was suspended in 100 mL of distilled water/kg feed and sprayed gradually onto the basal feed during continuous mechanical mixing. Mixing was continued before pelletizing to promote uniform distribution. The basal formulation, designed to provide approximately 39% crude protein in accordance with the nutritional requirements of common carp [78,79], consisted of fish meal (350 g/kg), soybean meal (300 g/kg), broken rice (140 g/kg), rice bran (90 g/kg), soybean oil (60 g/kg), tapioca starch (50 g/kg), and a vitamin premix (10 g/kg). The diets were thoroughly mixed, pelleted, dried at 45 °C for 48 h, cooled, sealed in polyethylene bags, protected from light using black outer bags, and stored at 4 °C. Fresh diets were prepared every two weeks throughout the feeding trial. Proximate composition was determined according to AOAC [80]. Total carotenoid content of the experimental diets was determined as described for *R. mucilaginosa* powder in Section 2.1, following the modified spectrophotometric method of Carvalho et al. [77]. The analyzed nutrient composition of the experimental diets is presented in Table 1.

### 2.3. Experimental Design and Feeding Trial

Juvenile fancy carp (*C. carpio*) were obtained from a commercial hatchery in Maha Sarakham Province, Thailand, and acclimated for two weeks in a 5 × 10 m concrete tank while being fed a commercial diet. After acclimation, healthy fish with an initial body weight of 21.26 ± 0.51 g and a total length of 11.06 ± 0.06 cm were randomly assigned to a completely randomized design comprising five dietary treatments with three replicate cages per treatment. Each treatment received one of the experimental diets containing 0, 0.5, 1, 2, or 4 g/kg *R. mucilaginosa* powder (RM0, RM0.5, RM1, RM2, and RM4, respectively). Fish were stocked at a density of 10 fish per 1 × 1 × 1 m cage (30 fish per treatment; 150 fish in total) and reared for 70 days under a natural photoperiod (12 h light:12 h dark) with continuous aeration. Fish were fed twice daily (08:00 and 16:00 h) at 5% of body weight per day, and feeding rates were adjusted biweekly based on bulk weight measurements. Approximately one-third of the rearing water was replaced every two weeks to limit the accumulation of suspended solids and metabolic wastes while minimizing abrupt environmental changes; additional water exchange was performed when required to maintain suitable water quality.

### 2.4. Growth Performance and Feed Utilization

Following the 70-day feeding trial, fish were fasted for 24 h before sampling to ensure consistent measurements. All fish from each cage were counted to determine survival rate (SR), and individual body weight and total length were recorded. These data were used to evaluate growth performance and feed utilization, including weight gain (WG), specific growth rate (SGR), average daily gain (ADG), feed conversion ratio (FCR), and survival rate (SR), using the standard equations described by Panchan et al. [81] as follows:

Weight gain (WG; g) = final body weight (g) − initial weight (g);
Specific growth rate (SGR; %/day) = 100 × [(ln final weight (g) − ln initial weight (g))/days];
Average daily gain (ADG; g/day) = [final weight (g) − initial weight (g)]/days;
Feed conversion ratio (FCR) = total feed (g)/weight gain (g);
Survival rate (SR, %) = [number of survived fish/initial number of fish] × 100.

### 2.5. Body Coloration and Tissue Carotenoid Analysis

At the end of the 70-day feeding trial, body coloration was evaluated using a portable colorimeter with 4 mm aperture (NR60CP, 3nh global; Guangzhou, China) at a standardized location on both sides of each fish, below the dorsal fin and above the lateral line. Three measurements were obtained from each location, and the mean values were used to determine the CIE Lab* color coordinates. Hue angle (H°) and chroma (C*) were subsequently calculated according to Hunt [82]. Following color assessment, three fish were randomly sampled from each replicate cage (nine fish per treatment), euthanized with an overdose of clove oil (50 mg/L), and skin, fin, and muscle tissues were collected for carotenoid analysis. Tissue samples from fish within each replicate were pooled, dried at temperatures below 50 °C, finely ground, and stored at −20 °C until analysis. Total carotenoid content of the skin, fin, and muscle samples was determined according to the method described in Section 2.1 [77].

### 2.6. Blood Collection and Serum Biochemical Analyses

At the end of the 70-day feeding trial, three fish were randomly sampled from each replicate cage (nine fish per treatment) and anesthetized with clove oil following Gokulakrishnan et al. [83]. Blood was collected from the caudal vein using sterile 3 mL syringes, pooled within each replicate, and divided into two aliquots. One aliquot was transferred into clot-activator tubes for serum biochemical and cortisol analyses, while the other was retained for antioxidant and innate immune assays. Serum was separated by centrifugation at 5000 rpm for 10 min and analyzed using an A15 Biochemistry Analyzer (Biosystems S.A., Barcelona, Spain). Aspartate aminotransferase (AST), alanine aminotransferase (ALT), and blood urea nitrogen (BUN) were determined using the IFCC kinetic method [84]. Glucose and cholesterol were measured enzymatically [85], whereas total protein and albumin were quantified by the colorimetric method of Lowry et al. [86]. Globulin concentration was calculated as the difference between total protein and albumin, and serum cortisol was determined using a chemiluminescent microparticle immunoassay (CMIA) with a Siemens Immulite^®^ 1000 analyzer (Siemens Healthcare Diagnostics, Tarrytown, NY, USA).

### 2.7. Digestive Enzymatic Activity

At the end of the 70-day feeding trial, digestive tissues were collected from each replicate cage (nine fish per treatment). Samples from each replicate were pooled, homogenized in 50 mM Tris-HCl buffer (pH 7.5), and centrifuged at 15,000 rpm for 5 min at 4 °C. The resulting supernatant was collected as the crude enzyme extract for enzymatic analyses. Protease, amylase, and lipase activities were determined according to the methods described by Bezerra et al. [87], Wangkahart et al. [88], and Iijima et al. [89], respectively.

### 2.8. Antioxidant Enzyme Activity and Malondialdehyde (MDA)

Serum samples were used to determine the activities of catalase (CAT), glutathione peroxidase (GPx), lysozyme (LZM), glutathione reductase (GRD), and myeloperoxidase (MPO). Serum was obtained by centrifugation at 10,000 rpm for 5 min, and enzyme activities were measured according to the methods described by Weydert and Cullen [90], Fontagné-Dicharry et al. [91], Ellis [92], and Sahoo et al. [93], respectively. All assays were performed in 96-well microplates, and absorbance was measured using a VersaMax™ microplate reader (Molecular Devices LLC, San Jose, CA, USA).

Hepatic lipid peroxidation was evaluated by measuring malondialdehyde (MDA) concentration according to the method of Maltez et al. [94]. Liver samples were homogenized, centrifuged, and reacted with thiobarbituric acid (TBA), and the absorbance of the resulting chromogen was measured at 532 nm.

### 2.9. Expression of Growth- and Immune-Related Genes

Liver samples were collected from three fish randomly selected from each replicate cage (nine fish per treatment) for quantitative real-time PCR (RT-qPCR) analysis. Total RNA was extracted using TRIzol™ Reagent (Invitrogen, Thermo Fisher Scientific, Waltham, MA, USA), purified, and reverse-transcribed into complementary DNA (cDNA) according to the methods described by Qi et al. [95] and Chen et al. [96]. Relative gene expression was quantified using a CFX Connect™ Real-Time PCR Detection System (Bio-Rad Laboratories, Hercules, CA, USA) with SYBR^®^ Green I (Invitrogen, Thermo Fisher Scientific, Waltham, MA, USA) and IMMOLASE™ DNA Polymerase (Bioline, London, UK). The expression levels of catalase (*CAT*), vitellogenin (*VTG*), glutathione peroxidase (*GPx*), heat shock protein 70 (*HSP70*), interleukin-1 beta (*IL-1β*), and superoxide dismutase (*SOD*) were normalized to the reference gene *β-actin*, and relative gene expression was calculated using the fold-change method, with the control group used as the calibrator. Gene-specific primer sequences (forward and reverse) were adopted from Prisingkorn et al. [24] and are presented in Table 2.

### 2.10. Intestinal Histomorphology and Liver Histopathology

Intestinal and liver tissues were collected from three fish randomly selected from each treatment for histological evaluation. Intestinal samples were fixed in 10% neutral-buffered formalin, dehydrated through a graded ethanol series, embedded in paraffin, sectioned at 5–7 μm, and stained with hematoxylin and eosin (H & E) and Polychrome. Intestinal morphometric parameters, including villus height, villus width, muscularis thickness, and goblet cell density, were quantified using LAS V4.5 software (Leica Microsystems, Wetzlar, Germany). For each fish, ten well-oriented, intact villi were randomly selected from non-consecutive sections, and villus height (µm) was measured from the tip of the villus to the villus crypt junction, while villus width (µm) and muscularis thickness (µm) were measured at the mid villus region. Goblet cells (cells/villus) were counted along one side of each of the ten selected villi, from the base to the tip, under a light microscope at 10× magnification and identified based on their characteristic flask or goblet shaped morphology with strong Polychrome/H&E staining that distinguished them from surrounding absorptive enterocytes. The mean number of goblet cells per villus was calculated for each fish and used for subsequent statistical analysis.

Liver tissues were processed for histopathological examination according to the procedures described by Roberts [97]. Representative sections were examined microscopically at 40× magnification to evaluate hepatic architecture and identify histopathological alterations, including hepatocellular vacuolation, necrosis, hemorrhage, and inflammatory cell infiltration.

### 2.11. Statistical Analysis

Data are expressed as the mean ± standard error (SE, *n* = 3). Normality and homogeneity of variance were assessed before statistical analysis, using the Shapiro–Wilk and Levene tests, respectively. Differences among dietary treatments were evaluated using one-way analysis of variance (ANOVA), followed by Duncan’s multiple range test for pairwise comparisons when significant effects were detected. Statistical significance was accepted at *p* < 0.05. All statistical analyses were performed using the SPSS statistics package software (version 29.0.1.0).

## 3. Results

### 3.1. Growth Performance and Feed Utilization

The effects of dietary *R. mucilaginosa* powder supplementation on growth performance, feed utilization, and survival are presented in Table 3. Significant treatment effects were observed for final body weight (FBW; *p* = 0.005), weight gain (WG; *p* = 0.010), average daily gain (ADG; *p* = 0.010), and feed conversion ratio (FCR; *p* < 0.001), whereas the remaining growth parameters were not significantly affected (*p* > 0.05). The RM0.5 group exhibited the highest FBW (45.95 ± 2.05 g), WG (25.38 ± 1.56 g), and ADG (0.36 ± 0.02 g/day), although these values did not differ significantly from those of the control or RM1 groups (*p* > 0.05). FCR was significantly lower in the RM0.5 (1.63 ± 0.03) and RM1 (1.70 ± 0.04) groups than in the control (1.87 ± 0.05), RM2 (2.07 ± 0.03), and RM4 (2.15 ± 0.01) groups (*p* < 0.001). Survival was 100% across all treatments.

### 3.2. Fish Body Coloration and Total Carotenoid Content

**Figure 1:**
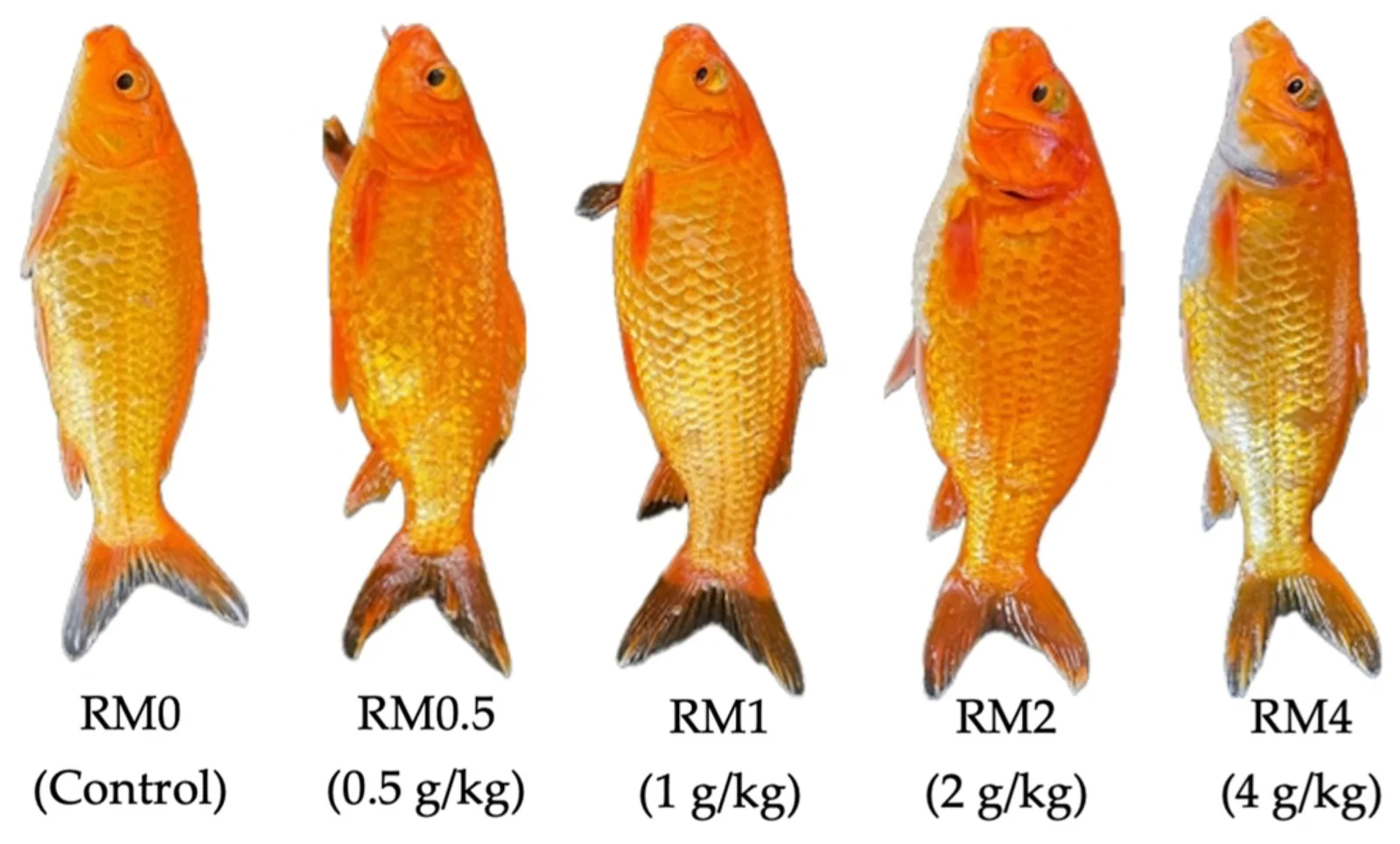
Body coloration of fancy carp after 70 days of dietary *R. mucilaginosa* powder supplementation.

The effects of dietary *R. mucilaginosa* powder supplementation on body coloration and tissue carotenoid deposition are summarized in Table 4. Baseline color parameters (L*, a*, b*, H°, and C*) did not differ among treatments (*p* = 0.899, 0.746, 0.961, 0.472, and 0.855, respectively), indicating comparable initial body coloration across all groups. After 70 days, all color parameters differed significantly (*p* < 0.001). The RM2 group exhibited the highest skin redness (a* = 17.94 ± 0.34), significantly exceeding the control (12.58 ± 0.51), RM0.5 (15.03 ± 0.26), RM1 (13.17 ± 0.33), and RM4 (13.78 ± 0.48) groups (*p* < 0.001). In contrast, the highest lightness (L*) was observed in the control (52.17 ± 0.45) and RM4 (51.82 ± 0.24), the highest yellowness (b*) in the control (48.56 ± 1.20), the highest hue angle (H°) in RM1 (23.12 ± 0.55), and the highest chroma (C*) in RM4 (48.83 ± 0.45), which was comparable to the control (47.92 ± 0.76) (*p* < 0.001). Visual observations confirmed the most intense orange coloration in the RM2 group (Figure 1). Tissue carotenoid concentrations also differed significantly (*p* < 0.001). RM2 showed the highest skin carotenoid content (15.62 ± 0.89 µg/g), the control had the highest fin carotenoid concentration (88.84 ± 2.98 µg/g), comparable to RM4 (84.81 ± 0.96 µg/g), and RM1 exhibited the highest muscle carotenoid concentration (1.67 ± 0.06 µg/g).

### 3.3. Blood Chemical Parameters

The serum biochemical parameters and cortisol concentrations of fancy carp after the 70-day feeding trial are presented in Table 5. No significant differences were observed among the experimental diets for any of the measured serum biochemical parameters, including total protein (*p* = 0.809), albumin (*p* = 0.387), globulin (*p* = 0.905), AST (*p* = 0.895), ALT (*p* = 0.822), blood urea nitrogen (*p* = 0.062), cholesterol (*p* = 0.711), and glucose (*p* = 0.709). Similarly, no significant difference in cortisol was observed among the five experimental diet groups (*p* = 0.188).

### 3.4. Digestive Enzymatic Activity

The effects of dietary *R. mucilaginosa* powder supplementation on digestive enzyme activities are presented in Table 6. Protease activity was not significantly affected by dietary treatments (*p* = 0.726). In contrast, amylase (*p* < 0.001) and lipase (*p* = 0.036) activities showed significant differences among treatments. Fish fed the RM0, RM0.5, and RM1 diets exhibited higher amylase activity (2.42 ± 0.02, 2.39 ± 0.02, and 2.44 ± 0.02 U/mg protein, respectively) than those fed the RM2 (2.28 ± 0.04) and RM4 (2.21 ± 0.01) diets. Lipase activity was lowest in the RM1 group (49.45 ± 2.94 U/mg protein) and highest in the control (68.53 ± 2.50 U/mg protein).

### 3.5. Antioxidant Enzyme Activity

**Figure 2:**
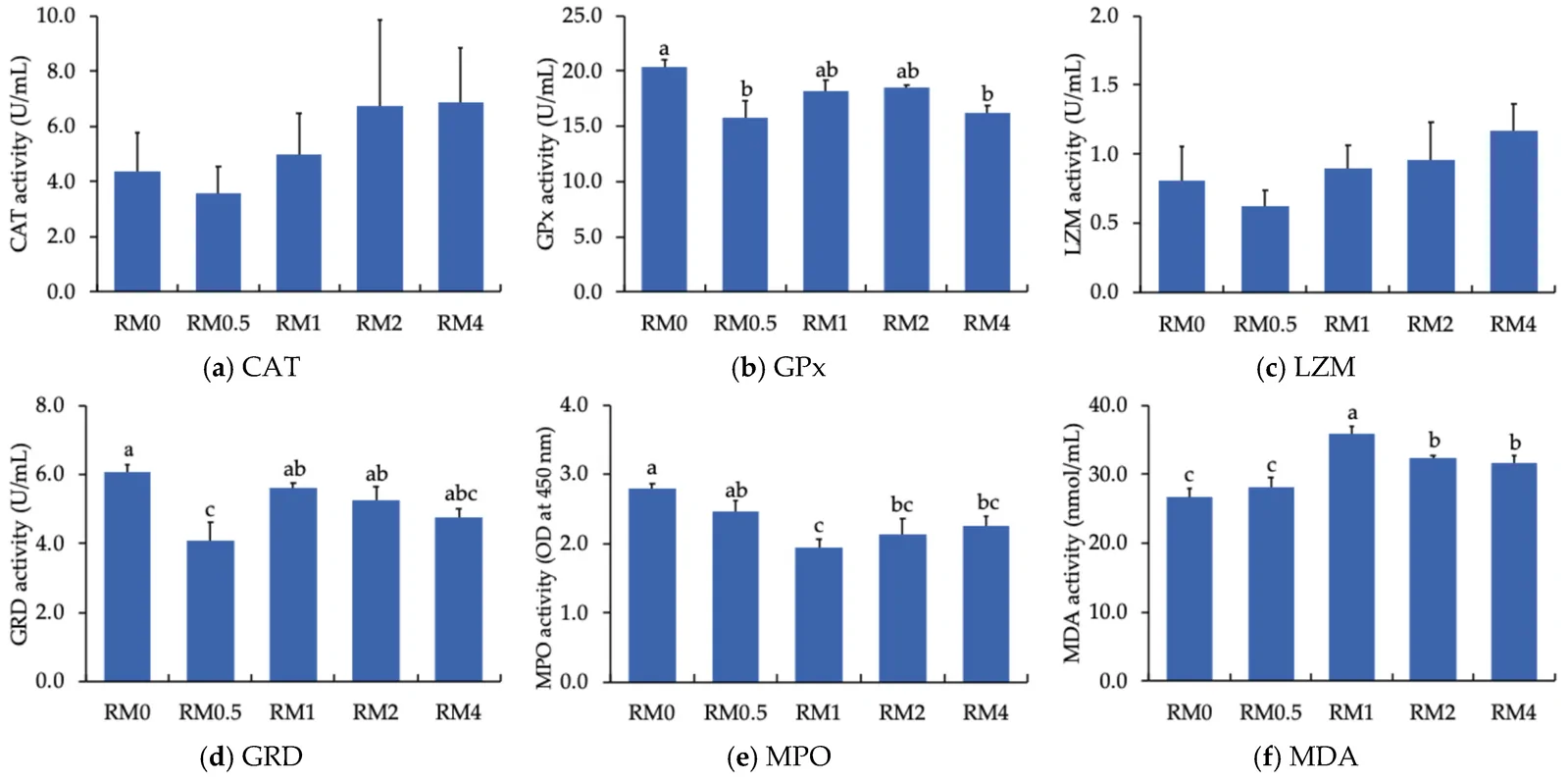
Serum antioxidant, innate immune responses and hepatic lipid peroxidation in fancy carp after 70 days of dietary *R. mucilaginosa* powder supplementation. Values are mean + SE (*n* = 8); different superscript letters indicate significant differences among treatments when the overall ANOVA was significant (*p* < 0.05); panels without letters did not show significant treatment effects. Abbreviations: CAT (Catalase activity), GPx (Glutathione peroxidase activity), LZM (Lysozyme activity), GRD (Glutathione reductase activity), MPO (Myeloperoxidase activity), and MDA (Malondialdehyde). RM0 (control), RM0.5 (0.5 g/kg), RM1 (1 g/kg), RM2 (2 g/kg), and RM4 (4 g/kg).

The effects of dietary *R. mucilaginosa* powder supplementation on antioxidant responses are shown in Figure 2. Catalase (CAT) and lysozyme (LZM) activities did not differ significantly among dietary treatments (*p* > 0.05). In contrast, significant differences were observed for glutathione peroxidase (GPx), glutathione reductase (GRD), myeloperoxidase (MPO), and hepatic malondialdehyde (MDA) (*p* < 0.05). GPx activity was highest in the control (RM0) group and was significantly lower in the RM0.5 and RM4 groups. Similarly, GRD activity reached its highest level in the control group, whereas the RM0.5 group exhibited the lowest activity. MPO activity was highest in the RM0 and RM0.5 groups and lowest in the RM1 group. Hepatic MDA concentration also differed significantly among treatments, with the highest level observed in the RM1 group, intermediate levels in the RM2 and RM4 groups, and the lowest concentrations in the RM0 and RM0.5 groups (*p* < 0.05).

### 3.6. Expression of Growth- and Immune-Related Genes

**Figure 3:**
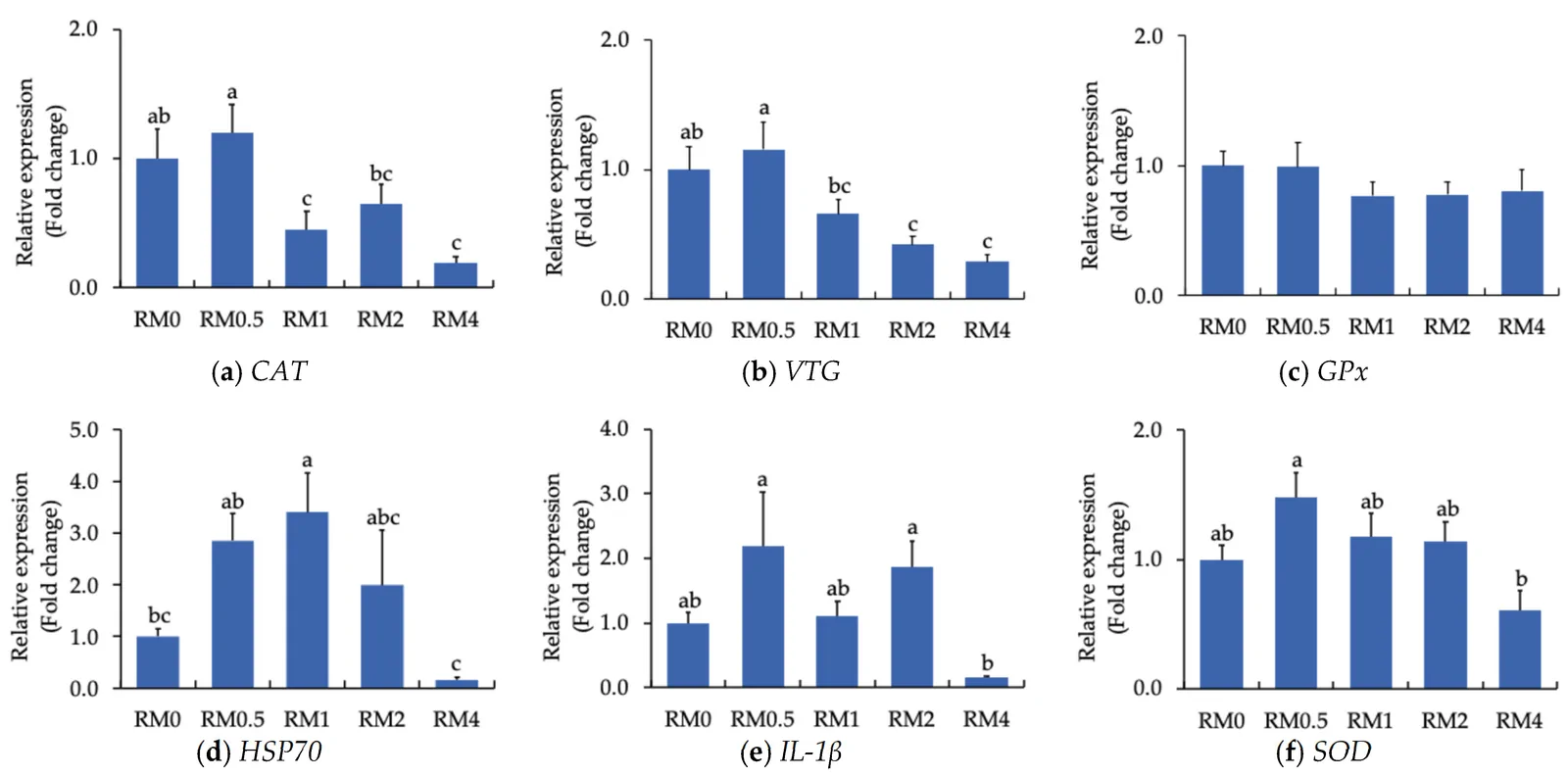
Relative hepatic expression of selected genes in fancy carp after 70 days of dietary *R. mucilaginosa* powder supplementation. Values are mean + SE (*n* = 4); Different superscript letters indicate significant differences among treatments when the overall ANOVA was significant (*p* < 0.05); panels without letters did not show significant treatment effects. Abbreviations: *CAT* (Catalase), *VTG* (Vitellogenin), *GPx* (Glutathione peroxidase), *HSP70* (Heat shock protein 70), *IL-1β* (Interleukin-1 beta), and *SOD* (Superoxide dismutase). RM0 (control), RM0.5 (0.5 g/kg), RM1 (1 g/kg), RM2 (2 g/kg), and RM4 (4 g/kg).

The relative hepatic expression of growth- and immune-related genes in response to dietary *R. mucilaginosa* supplementation is presented in Figure 3. Significant differences among dietary treatments were observed for *CAT, VTG, IL-1β, SOD*, and *HSP70* expression (*p* < 0.05), whereas *GPx* expression did not differ significantly among treatments (*p* > 0.05). Fish fed the RM0.5 diet exhibited the highest relative expression of *CAT, VTG, IL-1β*, and *SOD*, with expression levels significantly greater than those of the remaining dietary treatments (*p* < 0.05). In contrast, *HSP70* expression reached its highest level in the RM1 group and differed significantly from the other treatments (*p* < 0.05).

### 3.7. Intestinal Histomorphology and Liver Histopathology

**Figure 4:**
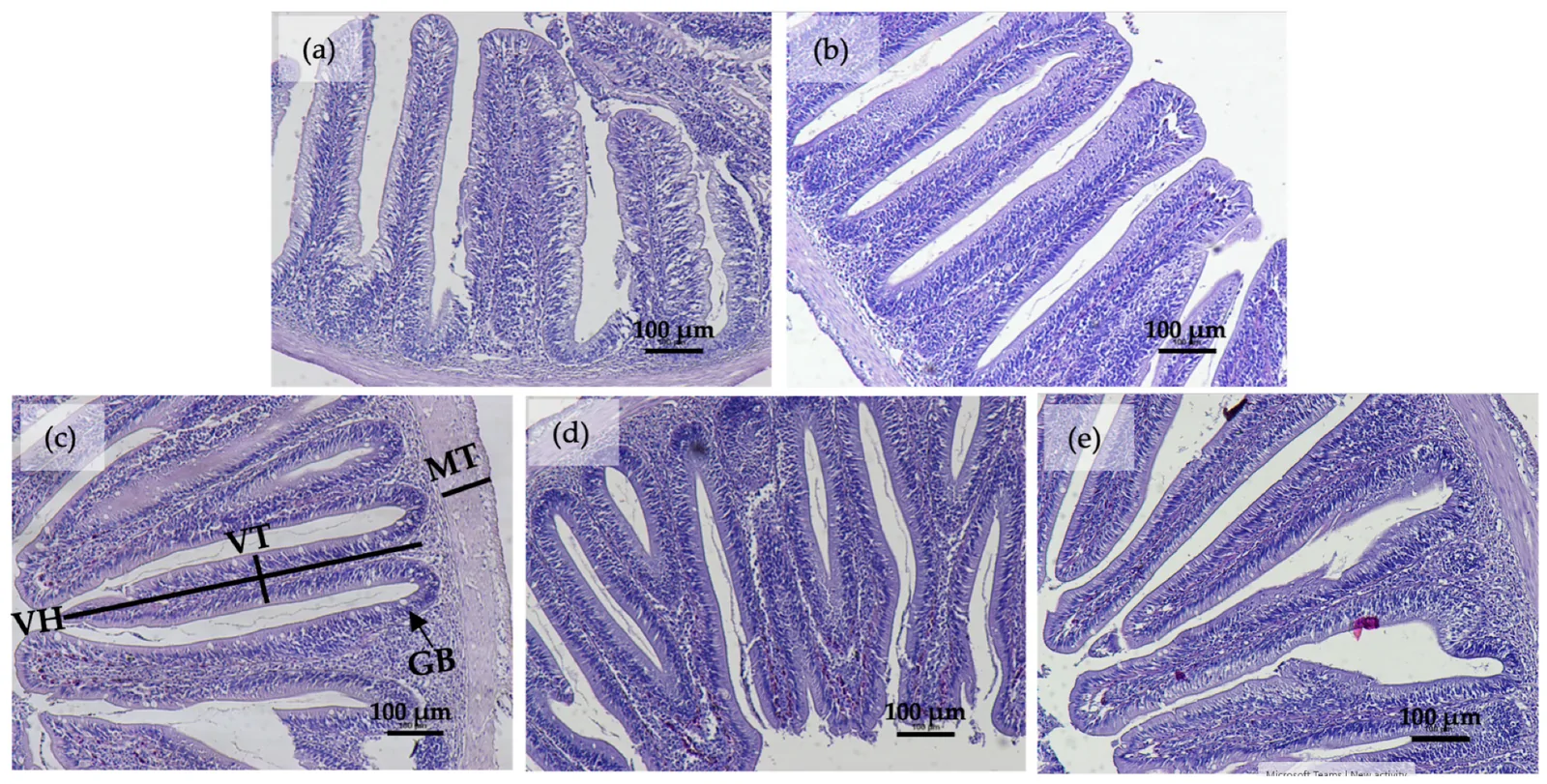
Histomorphological intestine section of fancy carp after 70 days of dietary *R. mucilaginosa* powder supplementation: (**a**) RM0 (control), (**b**) RM0.5 (0.5 g/kg), (**c**) RM1 (1 g/kg), (**d**) RM2 (2 g/kg), and (**e**) RM4 (4 g/kg). Abbreviations: MT, muscularis thickness; VH, villus height; VT, villus thickness; GB, goblet cells. Hematoxylin and eosin (H&E) staining. Scale bar = 100 µm. Magnification = 10×.

**Figure 5:**
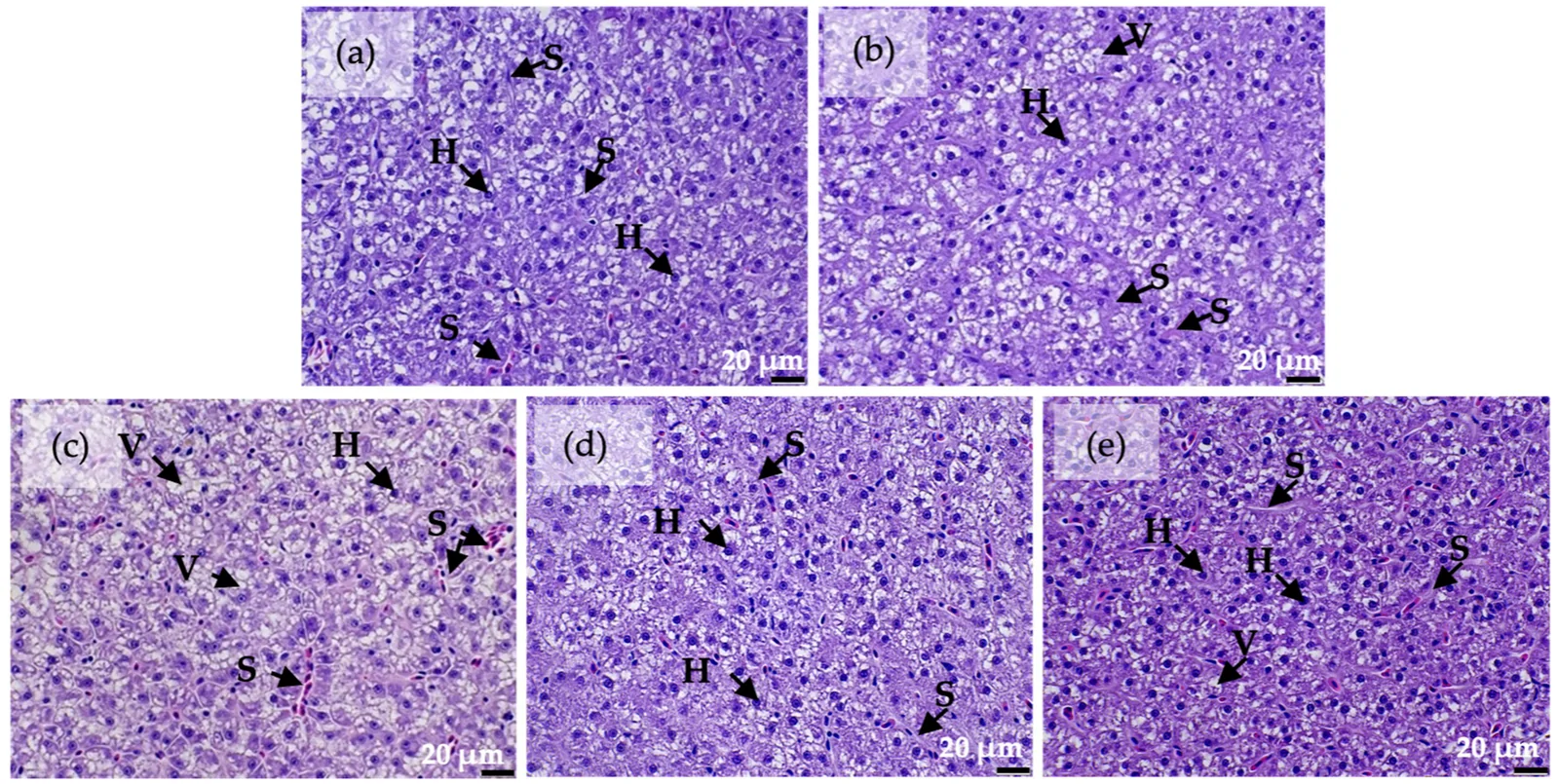
Histopathological liver sections of fancy carp after 70 days of dietary *R. mucilaginosa* powder supplementation: (**a**) RM0 (control), (**b**) RM0.5 (0.5 g/kg), (**c**) RM1 (1 g/kg), (**d**) RM2 (2 g/kg), and (**e**) RM4 (4 g/kg). Abbreviations: H (hepatocytes), S (sinusoids), and V (hepatocellular vacuolation). Hematoxylin and eosin (H&E) staining. Scale bar = 20 µm. Magnification = 40×.

The effects of dietary *R. mucilaginosa* powder supplementation on intestinal morphology are presented in Table 7 and Figure 4. Muscularis thickness, villus height, villus thickness, and goblet cell number differed significantly among treatments (all *p* < 0.001). The RM4 group exhibited the greatest muscularis thickness (91.81 ± 2.29 μm) and villus height (823.25 ± 5.24 μm), whereas the control showed the greatest villus thickness (95.30 ± 2.15 μm). Goblet cell number was highest in the RM1 group (15.50 ± 0.43 cells/villus) compared with the control (7.20 ± 0.33 cells/villus) (*p* < 0.001). Liver histology showed normal hepatic architecture with healthy hepatocytes, intact sinusoids, and no pathological alterations across all dietary treatments (Figure 5).

## 4. Discussion

The present study provides the first comprehensive evaluation of dietary *R. mucilaginosa* powder in fancy carp (*C. carpio*), assessing growth performance, pigmentation, physiological status, digestive enzyme activity, antioxidant and immune responses, gene expression, and tissue morphology. Overall, the responses were dose-dependent, with dietary supplementation at 0.5–2 g/kg providing the greatest biological benefits, whereas 4 g/kg offered no additional advantages and, in some cases, reduced performance. Beyond enhancing pigmentation, *R. mucilaginosa* significantly improved growth performance, feed utilization, digestive enzyme activity, gene expression, and intestinal morphology. The optimal inclusion level depended on the production objective: 0.5–1 g/kg maximized growth performance and feed efficiency, whereas 2 g/kg produced the greatest improvement in skin redness (CIE a*) and carotenoid deposition without compromising fish health. These findings support the use of dietary *R. mucilaginosa* to optimize either production efficiency or pigmentation in ornamental carp.

### 4.1. Low Dietary Inclusion Improved Growth Performance and Feed Utilization

One of the principal findings of the present study was that dietary supplementation with low levels of *R. mucilaginosa* (0.5–1 g/kg) improved feed utilization efficiency in fancy carp, as reflected by lower FCR and greater WG than those observed at higher inclusion levels. Although final body weight and several growth indices in the RM0.5 group were not consistently different from those of the control or RM1 groups, the significantly lower FCR indicates more efficient utilization of dietary nutrients rather than increased feed intake. Feed conversion ratio is widely recognized as a sensitive indicator of nutrient-use efficiency in common carp, and improvements in FCR often precede substantial differences in growth performance when dietary energy and nutrient utilization are optimized [79].

The improved feed efficiency at low supplementation levels is likely attributable to the combined effects of carotenoids, β-glucans, mannan-oligosaccharides (MOSs), proteins, vitamins, and other bioactive compounds present in *R. mucilaginosa* [48,49,50,53,54,55,61]. β-Glucans and MOSs are known to improve intestinal integrity and nutrient absorption, whereas carotenoids reduce oxidative stress through their antioxidant activity, allowing more nutrients to be allocated to growth rather than cellular maintenance [98].

These findings are consistent with studies in carp and other cultured fish. Rekha et al. [76] reported that dietary *Rhodotorula paludigena* improved growth performance in ornamental koi carp, whereas Mareš et al. [99] found that brewer’s yeast enhanced production performance in common carp without adverse physiological effects. Similar improvements in growth and feed utilization have also been reported in Nile tilapia fed *R. mucilaginosa* hydrolysate [67]. The comparable responses among these studies likely reflect the ability of yeast-derived functional ingredients to improve digestive efficiency and nutrient utilization. However, the absence of additional benefits at 4 g/kg indicates that the biological response reached a plateau, suggesting that moderate dietary inclusion is sufficient to maximize growth performance. Similar dose-dependent responses have been reported for yeast-based feed additives, in which moderate inclusion levels maximize biological benefits while higher levels provide little additional advantage [72,73]. Collectively, these results identify 0.5–1 g/kg *R. mucilaginosa* as the optimal inclusion range for improving feed efficiency and growth in fancy carp.

### 4.2. Moderate Supplementation Enhanced Pigmentation and Carotenoid Deposition

A major finding of the present study was that moderate dietary supplementation with *R. mucilaginosa* (2 g/kg) produced the greatest improvement in pigmentation, as evidenced by the highest skin redness (a*) and carotenoid accumulation. In contrast, lower supplementation levels (0.5–1 g/kg) produced superior growth but less intense pigmentation, indicating that carotenoid deposition requires a higher dietary inclusion level than growth optimization.

The enhanced pigmentation was closely associated with the significantly greater skin carotenoid concentration observed in the RM2 group, indicating more efficient carotenoid absorption, transport, and deposition in pigment cells. Improved intestinal morphology observed in the present study may also have facilitated carotenoid uptake, while enhanced antioxidant protection likely reduced pigment degradation, resulting in more stable and intense skin coloration. Together, these responses provide a plausible explanation for the superior pigmentation achieved at the moderate supplementation level.

These findings agree with previous studies demonstrating that carotenoid-producing microorganisms improve pigmentation, although optimal inclusion levels vary among species. Dietary supplementation with 5% *Rhodotorula paludigena* VA242 enhanced scale carotenoid concentration and pigmentation in ornamental koi carp [76], while 3% *R. mucilaginosa* increased skin brightness, red muscle pigmentation, and total carotenoids in gilthead seabream [75]. Likewise, alkali-treated *Rhodotorula dairenensis* SAG 17 improved integument pigmentation in tilapia and common carp [60], and various *R. mucilaginosa* JM-01 preparations enhanced shell pigmentation in *Penaeus vannamei*, with heat-killed cells producing the greatest response [70]. Consistent with these findings, 2 g/kg *R. mucilaginosa* produced the greatest carotenoid deposition and skin redness in fancy carp, whereas increasing supplementation to 4 g/kg provided no additional benefit, suggesting that carotenoid deposition had reached a physiological saturation point.

### 4.3. Blood Biochemical Responses Confirmed the Physiological Safety of Dietary R. mucilaginosa

An important finding of the present study was that dietary *R. mucilaginosa* improved growth performance and pigmentation without compromising physiological health. Serum biochemical parameters, including AST, ALT, BUN, total protein, albumin, globulin, glucose, cholesterol, and cortisol, were unaffected (*p* > 0.05), while fish maintained 100% survival and normal liver histology, indicating that supplementation up to 4 g/kg was safe over 70 days. The stable biochemical profile suggests that improved performance resulted from enhanced nutrient utilization rather than metabolic stress. These findings agree with Chen et al. [67] and Mareš et al. [99], who reported no significant changes in serum biochemical indices following dietary supplementation with *R. mucilaginosa*-derived hydrolyzed yeast or brewer’s yeast. Likewise, Liu et al. [72] found that marine red yeast improved metabolic homeostasis without impairing liver function, although greater changes in serum protein and lipid profiles were observed. In contrast, elevated cortisol concentrations were reported in *Atlantic salmon* fed a probiotic *Bacillus velezensis* V4 mixture containing *R. mucilaginosa* [69]; differences among studies likely reflect species, dietary formulations, and culture conditions. Generally, our findings suggest that *R. mucilaginosa* enhances growth and pigmentation without compromising metabolic homeostasis.

### 4.4. Digestive Enzyme Activity Reflected Differential Nutrient Utilization

Digestive enzyme responses provide a plausible physiological explanation for the improved feed utilization observed at low dietary inclusion levels of *R. mucilaginosa*. Fish fed 0.5–1 g/kg exhibited higher amylase activity, whereas protease activity remained unchanged, indicating that improved growth resulted primarily from enhanced carbohydrate digestion rather than increased protein hydrolysis [100]. The present findings are consistent with previous studies demonstrating that yeast-derived feed additives enhance digestive physiology. In common carp, dietary photosynthetic bacteria, *Bacillus* sp., or their combination (1 g/kg) improved growth, feed conversion ratio, and digestive enzyme activities, with the mixed probiotic producing the highest amylase and lipase activities [101]. Comparable responses were reported in juvenile sea cucumber fed *Rhodotorula benthica*, which exhibited increased amylase and other carbohydrate-degrading enzymes [58], and in animals receiving *Rhodotorula* sp. H26 plus *Bacillus* sp. BC26, which enhanced pepsin, trypsin, amylase, and lipase activities [65]. Similarly, dietary *S. cerevisiae* combined with *Tanacetum balsamita* essential oil stimulated amylase, lipase, and protease activities in Nile tilapia [102]. However, unlike these studies, only amylase activity increased in the present study, suggesting that *R. mucilaginosa* preferentially enhanced carbohydrate metabolism rather than broadly stimulating digestive enzyme secretion. This interpretation is supported by Tao et al. [103], who reported that 10 g/kg yeast culture improved intestinal morphology and digestive function in common carp, whereas excessive supplementation (30 g/kg) impaired enzyme activity and growth. Likewise, the reduced amylase activity at 2–4 g/kg indicates that digestive benefits are dose-dependent, with excessive supplementation providing no additional physiological advantage.

### 4.5. Antioxidant and Immune Responses Indicated Improved Physiological Resilience

In the present study, CAT and LZM activities remained unchanged, whereas GPx, GRD, MPO, and hepatic MDA showed significant but inconsistent dose-dependent responses. Notably, the lowest dietary inclusion level (0.5 g/kg) reduced hepatic MDA despite lower GPx and GRD activities, suggesting that oxidative stress was minimized without inducing glutathione-dependent antioxidant enzymes. In contrast, common carp fed *S. cerevisiae*, *Chlorella vulgaris*, or their combination exhibited increased CAT, GPx, and SOD activities with reduced MDA [104]. Similarly, hydrolyzed *R. mucilaginosa* enhanced antioxidant responses and reduced MDA in Nile tilapia [67], Pacific white shrimp fed 0.5% hydrolyzed *R. mucilaginosa* or its synbiotic combination with *Bacillus licheniformis* [68], GIFT tilapia receiving 0.53–0.60% marine red yeast [72], shrimp supplemented with 1% *Rhodosporidium paludigenum* [65], golden pompano fed 1–3% *R. mucilaginosa* [71], and Atlantic salmon receiving probiotic *R. mucilaginosa* [69]. These discrepancies likely reflect differences in host species, yeast preparation, inclusion level, experimental conditions, and health status. Most previous studies were conducted under disease or environmental challenges requiring antioxidant activation, whereas fish in the present study remained healthy, exhibited normal serum biochemical parameters and liver histology, and experienced minimal oxidative stress. Therefore, *R. mucilaginosa* likely maintained redox homeostasis rather than stimulating antioxidant enzyme production, with benefits primarily associated with improved intestinal function and nutrient utilization instead of inducing measurable antioxidant enzymatic responses under normal conditions.

### 4.6. Gene Expression Supported the Physiological Responses to Dietary Supplementation

Dietary *R. mucilaginosa* modulated hepatic gene expression in a dose-dependent manner, with 0.5 g/kg producing the highest expression of *CAT, SOD, IL-1β, and VTG*, whereas *HSP70* expression peaked at 1 g/kg, while *GPx* remained unchanged. These findings indicate that low dietary inclusion levels are sufficient to activate antioxidant and innate immune signaling without inducing broad stress responses. The upregulation of *CAT* and *SOD* suggests enhanced reactive oxygen species detoxification, while increased *IL-1β* likely reflects immune priming rather than excessive inflammation, consistent with the normal serum biochemistry and liver histology observed. Similarly, Prisingkorn et al. [24] reported that dietary 1% mulberry fruit extract upregulated hepatic *IL-1β, CAT*, and *VTG*, whereas *HSP70* expression reached its highest level at 2% supplementation, while *GPx* expression remained unaffected, mirroring the present findings. Although mulberry extract is a plant-derived additive rather than a microbial feed ingredient, the comparable transcriptional responses suggest that distinct functional feed additives can converge on common antioxidant and immune signaling pathways. Similar molecular responses have been reported in Nile tilapia, where 1% yeast hydrolysate increased intestinal IL-1β and antioxidant defenses [42], and in GIFT tilapia supplemented with approximately 0.5% marine red yeast, which upregulated *CAT, SOD*, and inflammatory cytokine genes [72]. Likewise, dietary R. *mucilaginosa* or *R. paludigena* enhanced *CAT, SOD, GPx, HSP70*, *IL-1β,* and other immune-related genes in red claw crayfish, Pacific white shrimp, and flowerhorn fish, thereby improving antioxidant capacity, immunity, intestinal health, and growth [63,64,68,73,74]. Together, these findings demonstrate that 0.5–1 g/kg *R. mucilaginosa* optimally regulates molecular pathways associated with antioxidant defense, immunity, and physiological performance without inducing excessive cellular stress.

### 4.7. Histological Observations Confirmed Intestinal Adaptation and Hepatic Safety

Fish fed 1–4 g/kg exhibited greater villus height, muscularis thickness, and goblet cell density, whereas liver histology remained normal across all treatments, demonstrating that supplementation was well tolerated despite pronounced intestinal remodeling. These findings agree with previous studies showing that yeast-derived feed additives consistently enhance intestinal structure while maintaining hepatic integrity. In common carp, dietary yeast fermentation products increased villus height and improved intestinal health without altering liver morphology [105], whereas *S. cerevisiae* and yeast cell wall prebiotics enhanced villus dimensions, muscular layer thickness, and crypt depth without inducing hepatic lesions [38]. Similar improvements in villus height and intestinal architecture have also been reported in Nile tilapia fed hydrolyzed *R. mucilaginosa* [67], Pacific white shrimp receiving hydrolyzed *R. mucilaginosa* or its synbiotic with *Bacillus licheniformis* [68], and red claw crayfish supplemented with 1.0 g/kg *R. mucilaginosa* [73]. These findings indicate that improved intestinal architecture is the principal mechanism responsible for the growth-promoting effects of dietary *R. mucilaginosa*, while normal liver histology confirms that these benefits are achieved without adverse hepatic effects at the tested inclusion levels.

## 5. Conclusions

This 70-day feeding trial demonstrated that dietary *Rhodotorula mucilaginosa* powder is a safe and effective multifunctional feed additive for fancy carp, with responses varying according to dietary inclusion level. Supplementation at 0.5–1 g/kg improved feed utilization and growth performance, whereas 2 g/kg produced the greatest enhancement in skin pigmentation and carotenoid deposition. These benefits were accompanied by favorable changes in digestive enzyme activity, intestinal morphology, hepatic gene expression, and physiological homeostasis, indicating that *R. mucilaginosa* functions not only as a natural pigment source but also as a functional ingredient that supports digestive efficiency, antioxidant and immune regulation, and overall fish health. Collectively, these findings demonstrate its potential as a sustainable alternative to synthetic pigment additives for ornamental aquaculture. Nevertheless, this study was conducted under controlled culture conditions and did not evaluate disease challenge, gut microbiota, carotenoid metabolic pathways, or long-term commercial performance. Future studies integrating microbiome and multi-omics approaches, together with long-term field trials and economic evaluations, are needed to clarify the underlying biological mechanisms and validate the practical application of *R. mucilaginosa* powder in sustainable ornamental fish production.

## Figures and Tables

**Table 1:** Proximate composition and carotenoid content of experimental diets supplemented with *R. mucilaginosa*.

Proximate Composition	RM0 (Control)	RM0.5 (0.5 g/kg)	RM1 (1 g/kg)	RM2 (2 g/kg)	RM4 (4 g/kg)
Dry matter (%)	97.54 ± 0.06	97.81 ± 0.05	96.84 ± 0.09	96.56 ± 0.01	97.34 ± 0.04
Crude protein (%)	39.06 ± 0.01	39.14 ± 0.29	39.34 ± 0.52	39.08 ± 0.42	39.09 ± 0.04
Crude lipid (%)	10.53 ± 0.04	10.79 ± 0.19	10.87 ± 0.06	10.84 ± 0.05	10.64 ± 0.01
Moisture (%)	2.46 ± 0.06	2.20 ± 0.05	3.16 ± 0.09	3.44 ± 0.01	2.67 ± 0.04
Ash (%)	11.4 ± 0.04	11.52 ± 0.00	11.55 ± 0.09	11.59 ± 0.16	11.49 ± 0.06
Crude fiber (%)	3.66 ± 0.02	3.64 ± 0.07	3.61 ± 0.04	3.57 ± 0.04	3.61 ± 0.04
Nitrogen-free extract (%) ^1^	32.89 ± 0.11	32.72 ± 0.04	31.48 ± 0.42	31.47 ± 0.35	32.51 ± 0.16
Gross energy (kcal/100 g) ^2^	461.45 ± 0.98	462.91 ± 0.58	465.47 ± 1.32	466.29 ± 0.06	466.99 ± 0.04
Digestible energy (kcal/100 g) ^3^	355.77 ± 0.22	357.44 ± 0.38	353.68 ± 0.38	352.56 ± 0.43	355.20 ± 0.58
Carotenoids content (µg/g dry weight)	5.35 ± 0.06	6.18 ± 0.32	6.30 ± 0.29	6.82 ± 0.26	6.95 ± 0.32

^1^ % Nitrogen-free extract = 100 − (% crude protein + % crude lipid + % ash + % crude fiber). ^2^ Gross energy (kcal/100 g) = (% crude protein × 4.0) + (% nitrogen-free extract × 2.5) + (% crude lipid × 8.0). ^3^ Digestible energy (kcal/100 g) = (% protein × 3.5) + (% fat × 9.44) + (% carbohydrate × 4.1). Values are presented as mean ± SE (dry matter basis).

**Table 2:** Primer sequences used for RT-qPCR analysis of target genes in fancy carp.

Gene Name	Nucleotide Sequence (5′ → 3′)	Product Length (bp)	Accession No.
Beta actin (*β-actin*)	Fw: CTGGTATCGTGATGGACTCT Rv: CAGAGCTTCTCCTTGATGTC	204	M24113
Catalase (*CAT*)	Fw: GAGCACGTAGGGTTCAAGTGC Rv: TCGCCTTATCTCTGTCTGCCA	162	NC_031745.1
Vitellogenin (*VTG*)	Fw: ATCTGAGATCCTCAGCCTGTGT Rv: TCACCAGACCCCCATCAATGTAA	107	NC_056618.1
Glutathione peroxidase (*GPx*)	Fw: TGTCCTTGATGGGTGATCCCA Rv: CAATGTCGCTGGTGAGGAACC	144	GQ376155.1
Heat shock protein 70 (*HSP70*)	Fw: GTTTTCCCTCCATCACTGACGA Rv: TTGATGATCCACAGGTGCAGT	70	NC_056599.1
Interleukin-1 beta (*IL-1β*)	Fw: CGAAGGCATAGGGCCTCTTA Rv: ACTATCGTAATCCATGCCGGT	100	NC_056581.1
Superoxide dismutase (*SOD*)	Fw: GATAGTGACAGACACGTCGGA Rv: AAGACTTTCGTCATTGCCTCC	180	XM_019111527

**Table 3:** Growth performance, feed utilization, and survival of fancy carp after 70 days of dietary *R. mucilaginosa* powder supplementation.

Parameter	RM0 (Control)	RM0.5 (0.5 g/kg)	RM1 (1 g/kg)	RM2 (2 g/kg)	RM4 (4 g/kg)	*p*-Value
IBW (g)	22.87 ± 1.73	20.57 ± 0.49	20.60 ± 1.07	21.97 ± 1.16	20.30 ± 0.89	0.478
FBW(g)	45.83 ± 0.31 ^a^	45.95 ± 2.05 ^a^	42.80 ± 1.47 ^ab^	41.72 ± 0.55 ^bc^	37.95 ± 0.89 ^c^	0.005
IL (cm)	11.17 ± 0.07	10.90 ± 0.12	11.17 ± 0.19	10.93 ± 0.12	11.13 ± 0.15	0.454
FL (cm)	13.93 ± 0.35	13.93 ± 0.35	13.93 ± 0.18	13.33 ± 0.15	13.73 ± 0.19	0.476
WG (g)	22.96 ± 1.91 ^ab^	25.38 ± 1.56 ^a^	22.20 ± 0.40 ^ab^	19.76 ± 1.03 ^bc^	17.65 ± 0.20 ^c^	0.010
SGR (%)	1.00 ± 0.12	1.15 ± 0.03	1.05 ± 0.03	0.92 ± 0.07	0.90 ± 0.03	0.112
ADG (g/day)	0.33 ± 0.03 ^ab^	0.36 ± 0.02 ^a^	0.32 ± 0.01 ^ab^	0.28 ± 0.01 ^bc^	0.25 ± 0.00 ^c^	0.010
FCR	1.87 ± 0.05 ^b^	1.63 ± 0.03 ^a^	1.70 ± 0.04 ^a^	2.07 ± 0.03 ^c^	2.15 ± 0.01 ^c^	<0.001
SR (%)	100	100	100	100	100	-

Values (mean ± SE, *n* = 3) in the same row followed by different superscripts are significantly difference at *p* < 0.05. Abbreviations: IBW (initial body weight), FBW (final body weight), IL (initial length), FL (final length), WG (weight gain), SGR (specific growth rate), ADG (average daily weight gain), FCR (feed conversion ratio) and SR (survival rate).

**Table 4:** Body color parameters and carotenoid content in the skin, fin, and muscle of fancy carp after 70 days of dietary *R. mucilaginosa* powder supplementation.

Parameter	RM0 (Control)	RM0.5 (0.5 g/kg)	RM1 (1 g/kg)	RM2 (2 g/kg)	RM4 (4 g/kg)	*p*-Value
Baseline L*	54.81 ± 3.62	52.47 ± 3.37	50.07 ± 2.15	52.61 ± 5.04	51.11 ± 1.40	0.899
L*	52.17 ± 0.45 ^a^	46.91 ± 0.10 ^c^	42.90 ± 0.44 ^d^	48.85 ± 0.26 ^b^	51.82 ± 0.24 ^a^	<0.001
Baseline a*	13.89 ± 0.47	10.31 ± 1.63	12.03 ± 1.30	12.67 ± 3.56	13.36 ± 1.60	0.746
a*	12.58 ± 0.51 ^c^	15.03 ± 0.26 ^b^	13.17 ± 0.33 ^c^	17.94 ± 0.34 ^a^	13.78 ± 0.48 ^c^	<0.001
Baseline b*	41.13 ± 6.51	39.65 ± 5.39	36.70 ± 2.41	41.11 ± 6.44	38.45 ± 1.78	0.961
b*	48.56 ± 1.20 ^a^	38.62 ± 0.87 ^b^	35.41 ± 0.87 ^c^	36.43 ± 0.24 ^bc^	46.52± 0.38 ^a^	<0.001
Baseline H°	67.32 ± 4.15	77.82 ±2.51	70.72 ±2.99	68.36 ± 6.98	70.08 ± 2.71	0.472
H°	14.55 ± 0.59 ^d^	21.84 ± 0.25 ^b^	23.12 ± 0.55 ^a^	21.73 ± 0.07 ^b^	17.60 ± 0.20 ^c^	<0.001
Baseline C*	44.95 ± 5.78	41.68 ±5.18	39.36 ± 2.41	44.98 ± 5.26	41.04 ± 1.18	0.855
C*	47.92 ± 0.76 ^a^	41.96 ± 1.12 ^b^	37.45 ± 0.48 ^c^	42.61 ± 0.70 ^b^	48.83 ± 0.45 ^a^	<0.001
**Total carotenoid (µg/g)**						
Skin	0.65 ± 0.17 ^c^	0.86 ± 0.07 ^c^	0.23 ± 0.06 ^c^	15.62 ± 0.89 ^a^	9.22 ± 0.72 ^b^	<0.001
Fin	88.84 ± 2.98 ^a^	70.60 ± 1.48 ^b^	32.73 ± 1.56 ^d^	54.30 ± 2.02 ^c^	84.81 ± 0.96 ^a^	<0.001
Muscle	0.90 ± 0.02 ^c^	0.89 ± 0.05 ^c^	1.67 ± 0.06 ^a^	1.30 ± 0.08 ^b^	1.43 ± 0.10 ^b^	<0.001

Values (mean ± SE, *n* = 3) in the same row followed by the different superscripts are significantly difference at *p* < 0.05.

**Table 5:** Serum biochemical parameters and cortisol concentrations of fancy carp fed diets containing graded levels of *R. mucilaginosa* powder for 70 days.

Parameter	RM0 (Control)	RM0.5 (0.5 g/kg)	RM1 (1 g/kg)	RM2 (2 g/kg)	RM4 (4 g/kg)	*p*-Value
Total protein (g/dL)	3.47 ± 0.07	3.37 ± 0.09	3.47 ± 0.26	3.20 ± 0.25	3.37 ± 0.09	0.809
Albumin (g/dL)	1.40 ± 0.06	1.43 ± 0.03	1.50 ± 0.10	1.30 ± 0.10	1.47 ± 0.03	0.387
Globulin (g/dL)	2.07 ± 0.03	1.93 ± 0.12	1.97 ± 0.19	1.90 ± 0.21	1.90 ± 0.06	0.905
AST (U/L)	165.33 ± 21.95	184.33 ± 20.73	160.67 ± 12.86	199.33 ± 51.81	166.33 ± 35.35	0.895
ALT (U/L)	60.33 ± 9.35	49.67 ± 1.33	48.00 ± 10.00	47.67 ± 8.82	50.67 ± 9.82	0.822
BUN (mg/dL)	2.67 ± 0.33	4.33 ± 0.33	3.33 ± 0.33	3.33 ± 0.33	3.33 ± 0.33	0.062
Cholesterol (mg/dL)	152.33 ± 20.22	138.67 ± 6.33	161.00 ± 21.00	134.67 ± 17.27	137.33 ± 2.85	0.711
Glucose (mg/dL)	71.33 ± 19.06	56.00 ± 6.93	86.00 ± 6.81	72.00 ± 19.86	74.67 ± 14.43	0.709
Cortisol (μg/dL)	46.53 ± 1.79	43.90 ± 3.36	45.80 ± 3.43	36.43 ± 4.00	46.60 ± 2.51	0.188

Data are given as mean ± SE (*n* = 3). Abbreviations: AST (Aspartate aminotransferase), ALT (Alanine aminotransferase), and BUN (Blood Urea Nitrogen).

**Table 6:** Digestive enzyme activities of fancy carp after 70 days of dietary *R. mucilaginosa* powder supplementation.

Parameter	RM0 (Control)	RM0.5 (0.5 g/kg)	RM1 (1 g/kg)	RM2 (2 g/kg)	RM4 (4 g/kg)	*p*-Value
Protease (U/mg protein)	20.01 ± 0.02	19.99 ± 0.01	20.00 ± 0.01	20.02 ± 0.03	20.01 ± 0.02	0.726
Amylase (U/mg protein)	2.42 ± 0.02 ^a^	2.39 ± 0.02 ^a^	2.44 ± 0.02 ^a^	2.28 ± 0.04 ^b^	2.21 ± 0.01 ^b^	<0.001
Lipase (U/mg protein)	68.53 ± 2.50 ^a^	63.62 ± 0.81 ^a^	49.45 ± 2.94 ^b^	65.71 ± 7.35 ^a^	65.37 ± 5.33 ^a^	0.036

Values (mean ± SE, *n* = 8) in the same row followed by different superscripts are significantly difference at *p* < 0.05.

**Table 7:** Intestinal morphometry of fancy carp after 70 days of dietary *R. mucilaginosa* powder supplementation.

Parameter	RM0 (Control)	RM0.5 (0.5 g/kg)	RM1 (1 g/kg)	RM2 (2 g/kg)	RM4 (4 g/kg)	*p*-Value
Muscularis thickness (µm)	56.51 ± 2.60 ^c^	57.49 ± 2.90 ^c^	74.10 ± 4.12 ^b^	74.36 ± 2.77 ^b^	91.81 ± 2.29 ^a^	<0.001
Villi height (µm)	558.09 ± 15.70 ^c^	543.45 ± 6.65 ^c^	661.17 ± 9.51 ^b^	651.52 ± 6.63 ^b^	823.25 ± 5.24 ^a^	<0.001
Villi thickness (µm)	95.30 ± 2.15 ^a^	73.98 ± 0.88 ^b^	61.53 ± 1.92 ^d^	69.39 ± 1.32 ^c^	64.83 ± 1.23 ^d^	<0.001
Goblet cell (cells/villus)	7.20 ± 0.33 ^d^	8.20 ± 0.33 ^d^	15.50 ± 0.43 ^a^	10.90 ± 0.38 ^c^	13.10 ± 0.31 ^b^	<0.001

Values (mean ± SE, *n* = 10) in the same row followed by different superscripts are significantly difference at *p* < 0.05.

## Data Availability

The datasets generated and/or analyzed during this study are available from the corresponding author upon reasonable request.

## References

[1] Santamaria Y.V., Santamaria W.C. (2011). Nutritional Requirements of Freshwater Ornamental Fish: A Review. Rev. MVZ Córdoba.

[2] Yang Z., Fu G., Lee M., Yeo S., Yue G.H. (2025). Genes for Editing to Improve Economic Traits in Aquaculture Fish Species. Aquac. Fish..

[3] Ninwichian P., Chookird D., Phuwan N. (2020). Effects of Dietary Supplementation with Natural Carotenoid Sources on Growth Performance and Skin Coloration of Fancy Carp, *Cyprinus carpio* L.. Iran. J. Fish. Sci..

[4] Lau C.C., Mohd Nor S.A., Tan M.P., Yeong Y.S., Wong L.L., Van de Peer Y., Sorgeloos P., Danish-Daniel M. (2023). Pigmentation Enhancement Techniques during Ornamental Fish Production. Rev. Fish Biol. Fish..

[5] Verlhac Trichet V., Amaya E. (2022). Astaxanthin Use as Carotenoid Source and Its Benefits in Feeds. Feed and Feeding Practices in Aquaculture.

[6] García-Chavarría M., Lara-flores M. (2013). The Use of Carotenoid in Aquaculture. Res. J. Fish. Hydrobiol..

[7] Maoka T. (2020). Carotenoids as Natural Functional Pigments. J. Nat. Med..

[8] Nakano T., Wiegertjes G. (2020). Properties of Carotenoids in Fish Fitness: A Review. Mar. Drugs.

[9] Pereira da Costa D., Campos Miranda-Filho K. (2020). The Use of Carotenoid Pigments as Food Additives for Aquatic Organisms and Their Functional Roles. Rev. Aquac..

[10] Elbahnaswy S., Elshopakey G.E. (2024). Recent Progress in Practical Applications of a Potential Carotenoid Astaxanthin in Aquaculture Industry: A Review. Fish Physiol. Biochem..

[11] de Carvalho C.C.C.R., Caramujo M.J. (2017). Carotenoids in Aquatic Ecosystems and Aquaculture: A Colorful Business with Implications for Human Health. Front. Mar. Sci..

[12] Saikia S., Das J.N. (2023). Carotenoids as a Dietary Supplement for Enhancing Pigmentation and Colouration in Ornamental Fishes: A Review. Uttar Pradesh J. Zool..

[13] Yaqoob S., Riaz M., Shabbir A., Zia-Ul-Haq M., Alwakeel S.S., Bin-Jumah M., Zia-Ul-Haq M., Dewanjee S., Riaz M. (2021). Commercialization and Marketing Potential of Carotenoids. Carotenoids: Structure and Function in the Human Body.

[14] Gupta S.K., Jha A.K., Pal A.K., Venkateshwarlu G. (2007). Use of Natural Carotenoids for Pigmentation in Fishes. Nat. Prod. Radiance.

[15] Sathyaruban S., Uluwaduge D.I., Yohi S., Kuganathan S. (2021). Potential Natural Carotenoid Sources for the Colouration of Ornamental Fish: A Review. Aquac. Int..

[16] Van Tran D., Tran T.L.T., Doan N.X., Dang T.T., Hua N.T., Pham H.Q. (2025). Comparative Impact of Synthetic and Natural Animal-Derived Carotenoids on Growth, Feed Utilization, and Pigment Enhancement in *Amphiprion ocellaris*. Fish. Aquat. Sci..

[17] Zhang J., Tian C., Zhu K., Liu Y., Zhao C., Jiang M., Zhu C., Li G. (2023). Effects of Natural and Synthetic Astaxanthin on Growth, Body Color, and Transcriptome and Metabolome Profiles in the Leopard Coralgrouper (*Plectropomus Leopardus*). Animals.

[18] Jiang J., Nuez-Ortin W., Angell A., Zeng C., de Nys R., Vucko M.J. (2019). Enhancing the Colouration of the Marine Ornamental Fish Pseudochromis Fridmani Using Natural and Synthetic Sources of Astaxanthin. Algal Res..

[19] Göçer M., Yanar M., Kumlu M., Yanar Y. (2006). The Effects of Red Pepper, Marigold Flower, and Synthetic Astaxanthin on Pigmentation, Growth, and Proximate Composition of *Penaeus semisulcatus*. Turk. J. Vet. Anim. Sci..

[20] Abbasi Ghadikolaei H., Kamali A., Soltani M., Sharifian M. (2017). Effects of Zingiber Officinale Powder on Growth Parameters, Survival Rate and Biochemical Composition of Body in Juvenile Common Carp (*Cyprinus carpio*). Iran. J. Fish. Sci..

[21] Kurnia A., Nur I., Muskita W.H., Hamzah M., Iba W., Patadjai R.S., Balubi A.M., Kalidupa N. (2019). Improving Skin Coloration of Koi Carp (*Cyprinus carpio*) Fed with Red Dragon Fruit Peel Meal. AACL Bioflux.

[22] Hekmatpour F., Nazemroaya S., Mousavi S.M., Amiri F., Feshalami M.Y., Sadr A.S., Mortezavizadeh S.A., Nejad L.M., Houshmand H., Kianersi F. (2023). Digestive Function and Serum Biochemical Parameters of Juvenile *Cyprinus carpio* in Response to Substitution of Dietary Soybean Meal with Sesame Seed (*Sesamum indicum*) Cake. Aquac. Rep..

[23] Le H.V., Pham L.V.B., Vo B.N.H., Le N.T.H., Nguyen Q.H., Luu P.V., Huynh T.D., Nguyen K.D., Nguyen N.H. (2024). Novel Application of Orange Marigold Flower Petal (*Tagetes erecta* L.) as a Dietary Supplement to Improve Skin Redness of Koi Carps (*Cyprinus carpio* L.). Sci. Technol. Dev. J. Eng. Technol..

[24] Prisingkorn W., Rinthong P., Kamolrat N., Wiriyapattanasub P., Wongmaneeprateep S., Suriyaphan J., Pholoeng A., Wangkahart E. (2025). Impact of Dietary Mulberry (*Morus alba*) Fruit Extract on the Growth Performance, Skin Color, and Immune Response of Fancy Carp (*Cyprinus carpio*). Aquac. Rep..

[25] Made P.S.S.A., Edi D.G.S., Rejeki I.G.A.D.S., Adnyana K.J.O., Sudiarta I.G., Janaguna I.M.A., Bergonio E.L., Sugiana I.P. (2026). Enhancement of Pigmentation in the Koi (*Cyprinus carpio*) Through Dietary Enrichment with Natural Carotenoid Sources. Egypt. J. Aquat. Biol. Fish..

[26] Van Het Hof K.H., West C.E., Weststrate J.A., Hautvast J.G.A.J. (2000). Dietary Factors That Affect the Bioavailability of Carotenoids. J. Nutr..

[27] Rodriguez-Amaya D.B., Esquivel P., Meléndez-Martínez A.J. (2023). Comprehensive Update on Carotenoid Colorants from Plants and Microalgae: Challenges and Advances from Research Laboratories to Industry. Foods.

[28] Daniel N., Sivaramakrishnan T., Subramaniyan S., Faizullah M.M., Fernando H. (2017). Application of Carotenoids on Coloration of Aquatic Animals. Int. J. Fish. Aquat. Res..

[29] Van Tran D., Luong H.T., Pham K.T., Dang T.T., Hua N.T., Pham H.Q. (2024). Plant-Based Carotenoid Supplementation: Growth, Feed Utilization Efficiency, and Coloration in False Clownfish (*Amphiprion ocellaris*). Isr. J. Aquac. Bamidgeh.

[30] Bogacz-Radomska L., Harasym J. (2018). β-Carotene-Properties and Production Methods. Food Qual. Saf..

[31] Xu S., Gao S., An Y. (2023). Research Progress of Engineering Microbial Cell Factories for Pigment Production. Biotechnol. Adv..

[32] Hoseinifar S.H., Maradonna F., Faheem M., Harikrishnan R., Devi G., Ringø E., Van Doan H., Ashouri G., Gioacchini G., Carnevali O. (2023). Sustainable Ornamental Fish Aquaculture: The Implication of Microbial Feed Additives. Animals.

[33] Zantioti C., Dimitroglou A., Mountzouris K.C., Miliou H., Malandrakis E.E. (2025). Use of Pigmented Fungi as Additives in Aquaculture. Aquac. Int..

[34] Mahdy M.A., Jamal M.T., Al-Harb M., Al-Mur B.A., Haque M.F. (2022). Use of Yeasts in Aquaculture Nutrition and Immunostimulation: A Review. J. Appl. Biol. Biotechnol..

[35] Martínez Cruz P., Ibáñez A.L., Monroy Hermosillo O.A., Ramírez Saad H.C. (2012). Use of Probiotics in Aquaculture. ISRN Microbiol..

[36] Mohammed E.A.H., Kovács B., Pál K. (2025). Recent Knowledge in the Application of *Saccharomyces cerevisiae* in Aquaculture: A Bibliometric and Narrative Review. Antibiotics.

[37] Agboola J.O., Øverland M., Skrede A., Hansen J.Ø. (2021). Yeast as Major Protein-rich Ingredient in Aquafeeds: A Review of the Implications for Aquaculture Production. Rev. Aquac..

[38] Mohammed E.A.H., Fehér M., Bársony P., Teye-Gaga C., Czeglédi L., Freytag C., Váradi A., Ahmed A.E.M., Pál K. (2026). Growth Performance, Gut Integrity and Intestinal Microbiome Responses of Juvenile Common Carp (*Cyprinus carpio* L.) to Probiotic and Prebiotic Supplementation. Animals.

[39] Van Doan H., Tapingkae W., Chaiyaso T., Wangkahart E., Panchan R. (2022). Effects of Red Yeast (*Sporidiobolus pararoseus*) on Growth, Innate Immunity, Expression of Immune—Related Genes and Disease Resistance of Nile Tilapia (*Oreochromis niloticus*). Probiotics Antimicrob. Proteins.

[40] Hao Q., Xia R., Zhang Q., Xie Y., Ran C., Yang Y., Zhou W., Chu F., Zhang X., Wang Y. (2022). Partially Replacing Dietary Fish Meal by *Saccharomyces cerevisiae* Culture Improve Growth Performance, Immunity, Disease Resistance, Composition and Function of Intestinal Microbiota in Channel Catfish (*Ictalurus punctatus*). Fish Shellfish Immunol..

[41] Islam S.M.M., Rohani M.F., Shahjahan M. (2021). Probiotic Yeast Enhances Growth Performance of Nile Tilapia (Oreochromis niloticus) through Morphological Modifications of Intestine. Aquac. Rep..

[42] Timothée Andriamialinirina H.J., Irm M., Taj S., Lou J.H., Jin M., Zhou Q. (2020). The Effects of Dietary Yeast Hydrolysate on Growth, Hematology, Antioxidant Enzyme Activities and Non-Specific Immunity of Juvenile Nile Tilapia, *Oreochromis niloticus*. Fish Shellfish Immunol..

[43] Long X., Wu X., Zhao L., Liu J., Cheng Y. (2017). Effects of Dietary Supplementation with *Haematococcus pluvialis* Cell Powder on Coloration, Ovarian Development and Antioxidation Capacity of Adult Female Chinese Mitten Crab, *Eriocheir sinensis*. Aquaculture.

[44] Li M., Wu W., Zhou P., Xie F., Zhou Q., Mai K. (2014). Comparison Effect of Dietary Astaxanthin and *Haematococcus pluvialis* on Growth Performance, Antioxidant Status and Immune Response of Large Yellow Croaker *Pseudosciaena crocea*. Aquaculture.

[45] Manklinniam P., Chittapun S., Maiphae S. (2018). Growth and Nutritional Value of *Moina macrocopa* (Straus, 1820) Fed with *Saccharomyces cerevisiae* and *Phaffia rhodozyma*. Crustaceana.

[46] Su F., Yu W., Liu J. (2020). Comparison of Effect of Dietary Supplementation with *Haematococcus pluvialis* Powder and Synthetic Astaxanthin on Carotenoid Composition, Concentration, Esterification Degree and Astaxanthin Isomers in Ovaries, Hepatopancreas, Carapace, Epithelium of Adult. Aquaculture.

[47] Li Z., Li C., Cheng P., Yu G. (2022). *Rhodotorula mucilaginosa*—Alternative Sources of Natural Carotenoids, Lipids, and Enzymes for Industrial Use. Heliyon.

[48] Ochoa-Viñals N., Alonso-Estrada D., Pacios-Michelena S., García-Cruz A., Ramos-González R., Faife-Pérez E., Michelena-Álvarez L.G., Martínez-Hernández J.L., Iliná A. (2024). Current Advances in Carotenoid Production by *Rhodotorula sp.*. Fermentation.

[49] Lucas D.C.R., Chisté R.C. (2025). Brazilian Biomes as Promising Resources of *Rhodotorula* Yeasts for the Biotechnological Production of Carotenoids. Chem. Biodivers..

[50] Díaz-Navarrete P., Marileo L., Madrid H., Mardones W., Correa Galeote D., Parra N., Dehnhardt-Amengual S., Dantagnan P. (2026). Native Oleaginous Yeasts *Rhodotorula mucilaginosa* and *Solicoccozyma gelidoterrea*: A Sustainable Biotechnological Alternative for Lipid Production with Potential Application in Diets for Farmed Fish. Front. Fungal Biol..

[51] Yun L., Wang W., Li Y., Xie M., Chen T., Hu C., Luo P., Li D. (2021). Potential Application Values of a Marine Red Yeast, *Rhodosporidiums sphaerocarpum* YLY01, in Aquaculture and Tail Water Treatment Assessed by the Removal of Ammonia Nitrogen, the Inhibition to *Vibrio spp*., and Nutrient Composition. PLoS ONE.

[52] Bogusławska-Wąs E., Dłubała A., Laskowska M. (2019). The Role of *Rhodotorula mucilaginosa* in Selected Biological Process of Wild Fish. Fish Physiol. Biochem..

[53] Ain Q.U., Baiju P., Rehman S., Chakraborty K., Pai A.A., Chandrasekar S., Ebeneezar S., Prabu D.L., Unnimaya T.D., Shylaja S. (2026). Polyphasic in Vitro Characterization of the Pigment-Producing Microfungus *Rhodotorula sp*. for Potential Application as a Probiotic in Mariculture. Front. Nutr..

[54] Sun M., Li D., Wang L., He Y., Hu Y., Yang M., Luo Z., Miao X., Yu S., Hua M. (2026). The Selenium-Enriched *Rhodotorula mucilaginosa* JAASRY1 Improved Oxidative Stress during the Aging Process via the Gut-Liver-Brain Axis. Front. Microbiol..

[55] Berezova K.A., Nikanova D.A., Artemyeva O.A., Kolodina E.N., Dovydenkova M.V. (2026). Influence of *Rhodotorula mucilaginosa* Yeast on the Microbiocenosis and Productive Qualities of Broiler Chickens. Agrar. Sci..

[56] Schafberg M., Loest K., Müller-Belecke A., Rohn S. (2020). Impact of Processing on the Antioxidant Activity of a Microorganism-Enriched Fish Feed and Subsequent Quality Effects on Fillets of Rainbow Trout (*Oncorhynchus mykiss*). Aquaculture.

[57] Ma Y., Li L., Li M., Chen W., Bao P., Yu Z., Chang Y. (2019). Effects of Dietary Probiotic Yeast on Growth Parameters in Juvenile Sea Cucumber, *Apostichopus japonicus*. Aquaculture.

[58] Wang J., Zhao L., Liu J., Wang H., Xiao S. (2015). Effect of Potential Probiotic *Rhodotorula benthica* D30 on the Growth Performance, Digestive Enzyme Activity and Immunity in Juvenile Sea Cucumber *Apostichopus japonicus*. Fish Shellfish Immunol..

[59] Yang Z.P., Sun J.M., Xu Z. (2015). Beneficial Effects of *Rhodotorula sp.* C11 on Growth and Disease Resistance of Juvenile Japanese Spiky Sea Cucumber *Apostichopus japonicus*. J. Aquat. Anim. Health.

[60] Ueno R., Hamada-Sato N., Ishida M., Urano N. (2011). Potential of Carotenoids in Aquatic Yeasts as a Phylogenetically Reliable Marker and Natural Colorant for Aquaculture. Biosci. Biotechnol. Biochem..

[61] Haldule S., Singhvi M., Zinjarde S. (2026). Rhodotorula and Phaffia: Pigment Producing Basidiomycetous Yeasts for Application in Aquaculture Practices. Arch. Microbiol..

[62] Nimrat S., Khaopong W., Sangsong J., Boonthai T., Vuthiphandchai V. (2021). Dietary Administration of Bacillus and Yeast Probiotics Improves the Growth, Survival, and Microbial Community of Juvenile Whiteleg Shrimp, *Litopenaeus vannamei*. J. Appl. Aquac..

[63] Kaewda J., Sangsawad P., Boonanuntanasarn S., Manassila P., Boontawan A., Ketudat-Cairns M., Sriphuttha C., Nakharuthai C. (2025). Effects of Probiotics Red Yeast *Rhodotorula paludigena* CM33 on Enhancing Color Pigmentation, Antioxidant Activity, Immune Response, Intestinal Microbiota, and Growth in a Commercial Ornamental Fish: Flowerhorn Fish. Aquac. Rep..

[64] Sriphuttha C., Limkul S., Pongsetkul J., Phiwthong T., Massu A., Sumniangyen N., Boontawan P., Ketudat-Cairns M., Boontawan A., Boonchuen P. (2023). Effect of Fed Dietary Yeast (*Rhodotorula paludigena* CM33) on Shrimp Growth, Gene Expression, Intestinal Microbial, Disease Resistance, and Meat Composition of *Litopenaeus vannamei*. Dev. Comp. Immunol..

[65] Yang S.P., Wu Z.H., Jian J.C., Zhang X.Z. (2010). Effect of Marine Red Yeast *Rhodosporidium paludigenum* on Growth and Antioxidant Competence of *Litopenaeus vannamei*. Aquaculture.

[66] Ma Y.X., Li L.Y., Bao P.Y., Li M., Chen W., Chang Y.Q. (2018). Effects of Combined Dietary Administration of *Rhodotorula Sp.* H26 and *Bacillus Sp.* BC26 on Growth, Immunity and Intestinal Microbiota in Juvenile Sea Cucumber, *Apostichopus japonicus*. Aquac. Res..

[67] Chen X.Q., Zhao W., Xie S.W., Xie J.J., Zhang Z.H., Tian L.X., Liu Y.J., Niu J. (2019). Effects of Dietary Hydrolyzed Yeast (*Rhodotorula mucilaginosa*) on Growth Performance, Immune Response, Antioxidant Capacity and Histomorphology of Juvenile Nile Tilapia (*Oreochromis niloticus*). Fish Shellfish Immunol..

[68] Chen M., Chen X.-Q., Tian L.-X., Liu Y.-J., Niu J. (2020). Enhanced Intestinal Health, Immune Responses and Ammonia Resistance in Pacific White Shrimp (*Litopenaeus vannamei*) Fed Dietary Hydrolyzed Yeast (*Rhodotorula mucilaginosa*) and *Bacillus licheniformis*. Aquac. Rep..

[69] Wang C., Liu Y., Sun G., Li X., Liu Z. (2019). Growth, Immune Response, Antioxidant Capability, and Disease Resistance of Juvenile Atlantic Salmon (*Salmo salar* L.) Fed *Bacillus velezensis* V4 and *Rhodotorula mucilaginosa* Compound. Aquaculture.

[70] Wang B., Liu Y., Luo K., Zhang S., Wei C., Wang L., Qiu Y., Tian X. (2023). ‘Biotic’ Potential of the Red Yeast *Rhodotorula mucilaginosa* Strain JM-01 on the Growth, Shell Pigmentation, and Immune Defense Attributes of the Shrimp, *Penaeus vannamei*. Aquaculture.

[71] Zhou C., Lin H., Xia D., Yang K., Yang Y., Huang Z., Wei Y. (2016). Effect of Dietary Marine Red Yeast *Rhodotorula mucilaginosa* on the Growth Performance, and Also Non-Specific Immune Responses of Juvenile Golden Pompano *Trachinotus ovatus* When Challenged with *Vibrio harveyi*. Isr. J. Aquac. Bamidgeh.

[72] Liu Y., Huang E., Li X., Xie Y., Tong T., Wang J., Zhang Q. (2024). Effects of Dietary Marine Red Yeast Supplementation on Growth Performance, Antioxidant, Immunity, Lipid Metabolism and MTOR Signaling Pathway in Juvenile Tilapia (*Oreochromis niloticus*). Aquac. Rep..

[73] Zhang Q., Liang Y., Li J., Li L., Meng L., Zeng Q., Wang D., Wang R., Tong T., Liu Y. (2025). *Rhodotorula mucilaginosa* Supplementation Could Significantly Affect the Growth Performance, Digestive Enzyme Activity, Antioxidant Capacity, Immune Function, and Intestinal Health in Red Claw Crayfish (*Cherax quadricarinatus*). Biology.

[74] Zhang Q., Meng L., Li J., Li L., Zeng Q., Wang R., Wang D., Tong T., Liu Y., Yang H. (2025). Effects of *Rhodotorula mucilaginosa* on Growth, Antioxidant, and Immune Function, and Toll/Imd and JAK-STAT Signaling Pathways in Red Claw Crayfish (*Cherax quadricanatus*). Aquac. Nutr..

[75] Zantioti C., Malandrakis E.E. (2026). Effects of Lyophilized Dietary Yeast *Rhodotorula mucilaginosa* on Skin and Fillet Pigmentation of Gilthead Seabream (*Sparus aurata*): A Computer-Based Image Analysis Assessment. Aquac. J..

[76] Rekha R., Nimsi K.A., Manjusha K., Sirajudheen T.K. (2024). Marine Yeast *Rhodotorula paludigena* VA 242 a Pigment Enhancing Feed Additive for the Ornamental Fish Koi Carp. Aquac. Fish..

[77] de Carvalho L.M.J., Gomes P.B., de Oliviera Godoy R.L., Pacheco S., do Monte P.H.F., de Carvalho J.L.V., Nutti M.R., Neves A.C.L., Vieira A.C.R.A., Ramos S.R.R. (2012). Total Carotenoid Content, α-Carotene and β-Carotene, of Landrace Pumpkins (*Cucurbita moschata* Duch): A Preliminary Study. Food Res. Int..

[78] Stankovic M., Dulic Z., Markovic Z. (2011). Protein Sources and Their Significance in Carp (*Cyprinus carpio* L.) Nutrition. J. Agric. Sci. Belgrade.

[79] Cho S.H., Jo J.Y., Kim D.S. (2001). Effects of Variable Feed Allowance with Constant Energy and Ratio of Energy to Protein in a Diet for Constant Protein Input on the Growth of Common Carp *Cyprinus carpio* L.. Aquac. Res..

[80] AOAC (1995). Official Methods of Analysis of the Association of Official Analytical Chemistry.

[81] Panchan R., Sutthi N., Wigraiboon S., Wangkahart E., Srinontong P., Thowanna C., Thumpala W., Yotsakun E. (2024). Chaya Leaf Meal as a Substitute for Soybean Meal in Climbing Perch (*Anabas testudineus* Bloch, 1792) Diet. Iran. J. Fish. Sci..

[82] Hunt R.W.G. (1977). The Specification of Colour Appearance. I. Concepts and Terms. Color Res. Appl..

[83] Gokulakrishnan M., Kumar R., Pillai B.R., Nanda S., Bhuyan S.K., Kumari R., Debbarma J., Ferosekhan S., Siddaiah G.M., Sundaray J.K. (2022). Dietary Brewer’s Spent Yeast Enhances Growth, Hematological Parameters, and Innate Immune Responses at Reducing Fishmeal Concentration in the Diet of Climbing Perch, *Anabas testudineus* Fingerlings. Front. Nutr..

[84] Henley K.S. (1980). International Federation of Clinical Chemistry (IFCC). Clin. Chim. Acta.

[85] Lott J.A., Turner K. (1975). Evaluation of Trinder’s Glucose Oxidase Method for Measuring Glucose in Serum and Urine. Clin. Chem..

[86] Lowry O.H., Rosebrough N.J., Farr A.L., Randall R.J. (1951). Protein Measurement with the Folin Phenol Reagent. J. Biol. Chem..

[87] Bezerra R.S., Lins E.J.F., Alencar R.B., Paiva P.M.G., Chaves M.E.C., Coelho L.C.B.B., Carvalho L.B. (2005). Alkaline Proteinase from Intestine of Nile Tilapia (*Oreochromis niloticus*). Process Biochem..

[88] Wangkahart E., Bruneel B., Wisetsri T., Nontasan S., Martin S.A.M., Chantiratikul A. (2022). Interactive Effects of Dietary Lipid and Nutritional Emulsifier Supplementation on Growth, Chemical Composition, Immune Response and Lipid Metabolism of Juvenile Nile Tilapia (*Oreochromis niloticus*). Aquaculture.

[89] Iijima N., Tanaka S., Ota Y. (1998). Purification and Characterization of Bile Salt-Activated Lipase from the Hepatopancreas of Red Sea Bream, *Pagrus major*. Fish Physiol. Biochem..

[90] Weydert C.J., Cullen J.J. (2010). Measurement of Superoxide Dismutase, Catalase and Glutathione Peroxidase in Cultured Cells and Tissue. Nat. Protoc..

[91] Fontagné-Dicharry S., Larroquet L., Dias K., Cluzeaud M., Heraud C., Corlay D. (2018). Effects of Dietary Oxidized Fish Oil Supplementation on Oxidative Stress and Antioxidant Defense System in Juvenile Rainbow Trout (*Oncorhynchus mykiss*). Fish Shellfish Immunol..

[92] Ellis A.E., Stolen J.S., Fletcher T.C., Anderson D.P., Roberson B.S., Van Muiswinkel W.B. (1990). Lysozyme Assays. Techniques in Fish Immunology.

[93] Sahoo P.K., Kumari J., Mishra B.K. (2005). Non-Specific Immune Responses in Juveniles of Indian Major Carps. J. Appl. Ichthyol..

[94] Maltez L.C., Barbas L.A.L., Nitz L.F., Pellegrin L., Okamoto M.H., Sampaio L.A., Monserrat J.M., Garcia L. (2018). Oxidative Stress and Antioxidant Responses in Juvenile Brazilian Flounder *Paralichthys orbignyanus* Exposed to Sublethal Levels of Nitrite. Fish Physiol. Biochem..

[95] Qi T., Wu Y., Fang H., Zhang F., Liu S., Zeng J., Yang J. (2022). Genetic Control of RNA Splicing and Its Distinct Role in Complex Trait Variation. Nat. Genet..

[96] Chen X.-J., Huang M.-Y., Wangkahart E., Cai J., Huang Y., Jian J., Wang B. (2023). Immune Response and Protective Efficacy of Mannosylated Polyethylenimine (PEI) as an Antigen Delivery Vector, Administered with a *Streptococcus agalactiae* DNA Vaccine in Nile Tilapia (*Oreochromis niloticus*). Fish Shellfish Immunol..

[97] Roberts R.J. (2012). Fish Pathology.

[98] Stavrakidis-Zachou O., Eding E.H., Papandroulakis N., Lika K. (2025). A Nutritional Bioenergetic Model for Farmed Fish: Effects of Food Composition on Growth, Oxygen Consumption and Waste Production. Aquac. Nutr..

[99] Mareš J., Poštulková E., Malý O., Zezula F., Šorf M., Všetičková L. (2023). Brewer’s Yeast as a Diet Supplement in Carp Aquaculture: Impact on Production Coefficients and Haematological and Biochemical Plasma Parameters. Ital. J. Anim. Sci..

[100] Krogdahl Å., Hemre G.I., Mommsen T.P. (2005). Carbohydrates in Fish Nutrition: Digestion and Absorption in Postlarval Stages. Aquac. Nutr..

[101] Yanbo W., Zirong X. (2006). Effect of Probiotics for Common Carp (*Cyprinus carpio*) Based on Growth Performance and Digestive Enzyme Activities. Anim. Feed Sci. Technol..

[102] Adineh H., Yousefi M., Al Sulivany B.S.A., Ahmadifar E., Farhangi M., Hoseini S.M. (2024). Effects of Dietary Yeast, *Saccharomyces cerevisiae*, and Costmary, *Tanacetum balsamita*, Essential Oil on Growth Performance, Digestive Enzymes, Biochemical Parameters, and Disease Resistance in Nile Tilapia, *Oreochromis niloticus*. Aquac. Nutr..

[103] Tao S., Wang J., Xue Z., Bai Y., Wang Y., Zhou W., Liang T., Yang Y. (2025). The Improved Growth Performance of *Cyprinus carpio* by Dietary Yeast Culture Depends on Improvement of Intestinal Structure and Digestive Enzyme Activities Rather than Changes of Intestinal Microbiota Composition. Aquac. Rep..

[104] El-Mashtoly M., Magouz F.I., Darwish S., Amer A.A., Zaineldin A.I., Gewaily M.S., Dawood M.A.O. (2024). Dietary *Chlorella vulgaris* and *Saccharomyces cerevisiae* Enhanced the Growth Performance, Blood Biomarkers, and Antioxidative Capacity of Common Carp (*Cyprinus carpio*). Sci. Afr..

[105] Wang M., Xia D., Yu L., Hao Q., Xie M., Zhang Q., Zhao Y., Meng D., Yang Y., Ran C. (2024). Effects of Solid-State Fermentation Product of Yeast Supplementation on Liver and Intestinal Health, and Resistance of Common Carp (*Cyprinus carpio*) against Spring Viraemia Carp Virus. Anim. Nutr..

